# *Xanthomonas oryzae* pv. *oryzae* TALE proteins recruit OsTFIIAγ1 to compensate for the absence of OsTFIIAγ5 in bacterial blight in rice

**DOI:** 10.1111/mpp.12696

**Published:** 2018-08-07

**Authors:** Wenxiu Ma, Lifang Zou, JI Zhiyuan, XU Xiameng, XU Zhengyin, Yangyang Yang, James R. Alfano, Gongyou Chen

**Affiliations:** ^1^ School of Agriculture and Biology Shanghai Jiao Tong University/Key Laboratory of Urban Agriculture by Ministry of Agriculture Shanghai 200240 China; ^2^ State Key Laboratory of Microbial Metabolism, School of Life Science & Biotechnology Shanghai Jiao Tong University Shanghai 200240 China; ^3^ The Center for Plant Science Innovation, University of Nebraska Lincoln NE 68588‐0660 USA; ^4^ Present address: Present address: National Key Facility for Crop Gene Resources and Genetic Improvement (NFCRI), Institute of Crop Science Chinese Academy of Agriculture Sciences (CAAS) Beijing 100081 China

**Keywords:** bacterial blight, *Oryza sativa*, OsTFIIAγ1, susceptibility, transcription activator‐like effector, *Xanthomonas oryzae* pv. *oryzae*

## Abstract

*Xanthomonas oryzae* pv. *oryzae* (*Xoo*), the causal agent of bacterial blight (BB) of rice, uses transcription activator‐like effectors (TALEs) to interact with the basal transcription factor gamma subunit OsTFIIAγ5 (Xa5) and activates the transcription of host genes. However, how OsTFIIAγ1, the other OsTFIIAγ protein, functions in the presence of TALEs remains unclear. In this study, we show that OsTFIIAγ1 plays a compensatory role in the absence of Xa5. The expression of *OsTFIIAγ1*, which is activated by TALE PthXo7, increases the expression of host genes targeted by avirulent and virulent TALEs. Defective *OsTFIIAγ1* rice lines show reduced expression of the TALE‐targeted susceptibility (*S*) genes, *OsSWEET11* and *OsSWEET14*, which results in increased BB resistance. Selected TALEs (PthXo1, AvrXa7 and AvrXa27) were evaluated for interactions with *OsTFIIAγ1*, Xa5 and xa5 (naturally occurring mutant form of Xa5) using biomolecular fluorescence complementation (BiFC) and microscale thermophoresis (MST). BiFC and MST demonstrated that the three TALEs bind Xa5 and OsTFIIAγ1 with a stronger affinity than xa5. These results provide insights into the complex roles of OsTFIIAγ1 and OsTFIIAγ5 in TALE‐mediated host gene transcription.

## Introduction

Bacterial plant pathogens reduce the yield of many important crops of global importance, including rice, tomatoes, peppers and citrus. *Xanthomonas* is a widespread bacterial genus that contains approximately 30 pathogenic species known to cause disease in over 300 plant hosts (Boch *etal*., 2014; Schornack *etal*., 2013). One particularly important pathogen within the genus *Xanthomonas* is *X. oryzae* pv. *oryzae* (*Xoo*), which causes bacterial blight (BB) of rice, a devastating disease in rice production areas.

Many *Xanthomonas* spp. cause plant disease by injecting transcription activator‐like effectors (TALEs) directly into plant host cells via the Type III secretion system (T3SS) (Chen *etal*., 2010; Mak *etal*., 2013). TALEs are then translocated to the nucleus where they bind to specific promoter sequences in host genes, which are designated as TAL effector‐binding elements (EBEs) (Chen *etal*., 2010; Mak *etal*., 2013). The DNA‐binding domain of TALEs consists of repeat variable diresidues (RVDs) that bind to a predictable DNA recognition code in the promoter of the TALE gene target (Boch *etal*., 2009; Moscou and Bogdanove, 2009). TALE‐like proteins are not restricted to the genus *Xanthomonas*, as they are found in other plant pathogens and endosymbionts, including *Ralstonia solanacearum* and *Burkholderia rhizoxinica*, respectively (de Lange *etal*., 2014). Apart from their EBE‐binding ability, it remains unclear how TALEs function to promote the transcription of target genes cooperatively with other transcriptional factors.

Rice has developed an innate immune system to detect invading pathogens and trigger defensive responses to neutralize infection. As a counter‐offensive strategy, *Xoo* can deploy several different methods to interfere with the rice defence response. These include the use of interfering TALEs (iTALES) or truncated TALEs (truncTALEs), which can disrupt *Xa1*‐mediated defences that are triggered by archetypal TALEs (Ji *etal*., 2016; Read *etal*., 2016). Futhermore, *Xoo* can also deploy TALEs that promote the transcription of susceptibility (*S*) genes in the *SWEET* gene family (Streubel *etal*., 2013; Zhou *etal*., 2015). SWEET proteins are responsible for sugar transport in rice and their production can foster pathogen growth (Chen, 2014; Chen *etal*., 2010).

In response to TALEs and other *Xoo* virulence strategies, rice has co‐evolved counter‐measures, such as the utilization of recessive resistance (*R*) genes for many of the *S* gene targets. These recessive *R* genes can result in TALE mistargeting, reduced TALE binding and increased plant disease resistance (Boch *etal*., 2014; Hutin *etal*., 2015a). Three recessive *R* genes (*xa13*, *xa25* and *xa41(t)*) have been identified in several rice varieties and are the EBE‐mutational alleles of *OsSWEET11*, *OsSWEET13* and *OsSWEET14*, respectively (Chu *etal*., 2006; Hutin *etal*., 2015b; Liu *etal*., 2011; Yang *etal*., 2006; Zhou *etal*., 2015). Furthermore, some rice plants utilize a strategy that allows TALEs to recognize the promoters of dominantly inherited executor *R* genes, which trigger TAL effector‐triggered immunity (ETI) (Boch *etal*., 2014; Zhang *etal*., 2015). Executor *R* gene products have been divided into two groups (Zhang *etal*., 2015). Members of group 1 function in plant development and physiology; this group includes BS3, an R protein from pepper that belongs to the flavin mono‐oxygenase family (Expósito‐Rodríguez *etal*., 2011; Romer *etal*., 2007). Group 2 contains R proteins from rice, including XA10, XA27 and XA23, which are activated by the cognate effectors (AvrXa10, AvrXa27 and AvrXa23) (Gu *etal*., 2005; Tian *etal*., 2014; Wang *etal*., 2015). XA10, localized to endoplasmic reticulum (ER), is associated with ER Ca^2+^ cation depletion (Tian *etal*., 2014) and shares 50% identity with XA23 (Wang *etal*., 2015). In contrast, XA27‐mediated resistance depends on localization to the apoplast (Wu *etal*., 2008). Interestingly, these *R* genes have no obvious relationship with known *R* or *S* genes, suggesting that further complex defence responses are in play.

Several important studies have been conducted to understand how *Xoo* uses its collection of TALEs to activate plant transcriptional factors and modulate plant defence. Sugio *etal*. (2007) described how the rice gene *OsTFX1*, which encodes a bZIP transcription factor, was targeted by the TALE PthXo6 from *Xoo* strain PXO99^A^. In the same study, these authors also showed that PthXo7 induces the expression of the transcription factor *OsTFIIAγ1* during rice infection by *Xoo* PXO99^A^ (Sugio *etal*., 2007). Interestingly, in addition to *OsTFIIAγ1*, rice contains another gene, *Xa5* (*OsTFIIAγ5*), that encodes the small (γ) subunit of the conserved general transcription factor TFIIA, which is important for polymerase II (Pol II)‐dependent transcription (Hoiby *etal*., 2007; Jiang *etal*., 2006). Recently, Xa5 and TFIIAγ proteins from rice, citrus, pepper and tomato have been shown to interact directly with a transcription factor binding (TFB) region in TALEs (Huang *etal*., 2017; Yuan *etal*., 2016). This is consistent with the hypothesis that TALEs may function as transcriptional activators by their involvement in the assembly of the transcription initiation complex at their target sites in plants (Boch and Bonas, 2010). However, information is lacking on how TALEs might specifically interact with the plant transcriptional machinery to modulate expression.

However, the rice recessive gene *xa5*, which is a natural allele of *Xa5*, contains a mutation in the 39th residue, in which the valine (V) residue is replaced with glutamine (E) (V39E) (Iyer and McCouch, 2004). It has been speculated that the missense mutation in *xa5* may confer resistance by abolishing the interaction between DNA‐associated TALEs and the preinitiation complex, which could attenuate the transcription of TALE‐targeted genes (Schornack *etal*., 2006, 2013). Indeed, Yuan *etal*. (2016) reported that xa5 fails to interact with several tested TALEs. Furthermore, TALE‐mediated induction of *R* or *S* genes is attenuated in the *xa5* background (Gu *etal*., 2009; Huang *etal*., 2016; Tian *etal*., 2014). However, there is no evidence supporting or negating the involvement of OsTFIIAγ1 in the assembly of the transcription initiation complex in rice plants.

To gain further insights into the fundamental roles of Xa5, xa5 and, especially, OsTFIIAγ1 in BB, we expressed *avrXa7*, *pthXo1* and *avrXa27* in *Xoo* strains PH and PE, which are *tal*‐free and *pthXo7*‐containing strains derived from PXO99^A^ (Ji *etal*., 2016). These strains were evaluated for pathogenicity in rice lines IR24 (*Xa5* and *OsTFIIAγ1*), IRBB5 (*xa5* and *OsTFIIAγ1*), *TF1* (*xa5* and inactive *OsTFIIAγ1*), DH (*Xa27*, *xa5* and *OsTFIIAγ1*) and 78‐1‐5 (*Xa27*, *Xa5* and *OsTFIIAγ1*). The interaction and affinities of Xa5, xa5 and OsTFIIAγ1 with *avrXa7*, *pthXo1* and *avrXa27* were also examined. The results suggest that OsTFIIAγ1 has a role in BB and compensates for the absence of Xa5.

## Results

### IRBB5‐incompatible *Xoo* strains are unable to activate *OsTFIIAγ1* expression

**Figure 1:**
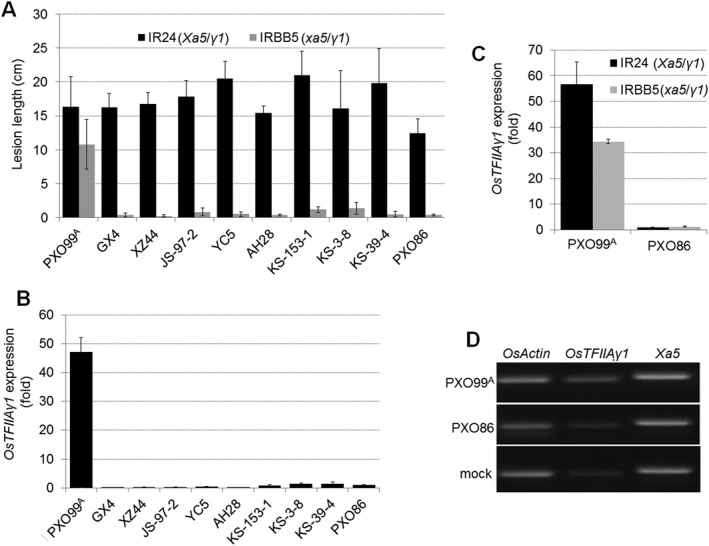
Virulence (lesion length) and *OsTFIIAγ1* expression levels during *Xanthomonas oryzae* pv. *oryzae* (*Xoo*) infection in IR24 and IRBB5 rice lines. (A) Lesion lengths induced by *Xoo* PXO99^A^, PXO86 and eight Chinese strains (GX4, XZ44, JS‐97‐2, YC5, AH28, KS‐153‐1, KS‐3–8, KS‐39‐4) in IR24 and IRBB5 rice. Bacteria were inoculated by tip‐cutting and lesion lengths were measured at 14 days post‐inoculation (dpi). The mean lesion lengths ± standard deviation (SD) (*n* = 5) are shown. (B) *OsTFIIAγ1* expression in IR24 rice seedlings inoculated with different *Xoo* strains. The expression of *OsTFIIAγ1* was evaluated by quantitative real‐time reverse transcription‐polymerase chain reaction (qRT‐PCR) at 24h post‐infiltration (hpi). (C) qRT‐PCR analysis of *OsTFIIAγ1* expression in IR24 and IRBB5 rice inoculated with *Xoo* PXO99^A^ and PXO86 at 24 hpi. Values in (B) and (C) represent the mean ± SD (*n* = 3). (D) RT‐PCR analysis of *OsTFIIAγ1* and *Xa5* transcription in IR24 seedlings 24h after infection with PXO99^A^, PXO86 and water (mock control). The result shown is representative of three replicates. *OsActin* was used as the reference gene in both RT‐PCR and qRT‐PCR.

To investigate whether naturally occurring *Xoo* strains isolated from the environment have the ability to evade *xa5*‐mediated resistance, we examined the virulence of 65 *Xoo* strains isolated from 13 rice‐planting provinces in China. The well‐characterized Philippine strains PXO99^A^ and PXO86 were included for comparative purposes (Table S1, see Supporting Information). The pathogenicity of *Xoo* strains was assessed in two near‐isogenic lines of rice, IR24 (*Xa5*) and IRBB5 (containing *xa5* in the IR24 background). *Xoo* strains were inoculated using a tip‐cutting method, and the lesion length was measured at 14 days post‐inoculation (dpi) (see Experimental procedures). The 65 Chinese isolates of *Xoo* were pathogenic in IR24 rice, but were incompatible in *xa5*‐containing rice IRBB5. Lesions caused by eight of the 65 strains in both IR24 and IRBB5 are shown (Fig. 1A). We observed that PXO99^A^ was compatible and PXO86 incompatible in IRBB5, which is consistent with previous results (Sugio *etal*., 2007). Previous work has shown that *OsTFIIAγ1* expression is activated by the TALE PthXo7, which is present in PXO99^A^, but absent in PXO86 (Sugio *etal*., 2007). Therefore, we used quantitative real‐time reverse transcription‐polymerase chain reaction (qRT‐PCR) to examine whether the expression of *OsTFIIAγ1* was altered during challenge with these *Xoo* strains. *OsTFIIAγ1* expression was activated in IR24 rice inoculated with *Xoo* PXO99^A^, but not with PXO86 or the eight Chinese *Xoo* strains (Fig. 1B). These results suggest that the Chinese isolates lack *pthXo7*, which is the case with PXO86.

PXO99^A^ caused significantly more disease in IR24 (lesion length, 16.3cm) than in IRBB5 (lesion length, 10.8cm) rice at 14 dpi (Fig. 1A). The reduced disease symptoms in the IRBB5 line could be partially due to *xa5* (mutant form of *Xa5*). It is also important to note that lesions in the PXO99^A^/IRBB5 interaction might be modulated in part by OsTFIIAγ1, which is activated by PthXo7 in PXO99^A^, but not in the other *Xoo* strains. To confirm this possibility, we compared the expression of *OsTFIIAγ1* in IR24 and IRBB5 by qRT‐PCR and RT‐PCR*. Xoo* PXO99^A^ induced the expression of *OsTFIIAγ1* in IRBB5, although the expression level was lower than in IR24 (Fig. 1C). Intriguingly, *OsTFIIAγ1* expression in IR24 and IRBB5 seedlings was significantly lower than that of *Xa5* (Fig. 1D) or *xa5* (Fig. S1, see Supporting Information). Taken together, the lesion lengths caused by PXO99^A^ in IRBB5 may be partially a result of the role of activated *OsTFIIAγ1* in the presence of *Xoo* TALEs.

### Activated *OsTFIIAγ1* enhances the expression of TALE targets in rice

The availability of a set of PXO99^A^‐derived strains that are lacking specific *tal* genes (Ji *etal*., 2016) enabled the examination of potential overlapping functions for *OsTFIIAγ1* and *xa5* in rice during challenge with PthXo7 and other selected TALEs. In our experiments, two PXO99^A^‐derived strains were utilized (Fig. S2, see Supporting Information): *Xoo* PH (lacks genes encoding known TALEs) and *Xoo* PE; the latter strain contains *pthXo7*, which activates the expression of *OsTFIIAγ1* (Fig. S2C). *Xoo* PH and PE were used to overexpress *pthXo1*, resulting in strains PH(*pthXo1*) and PE(*pthXo1*), as described in Methods S1 and S2, Figs S2 and S3, and Table S1 (see Supporting Information).

**Figure 2:**
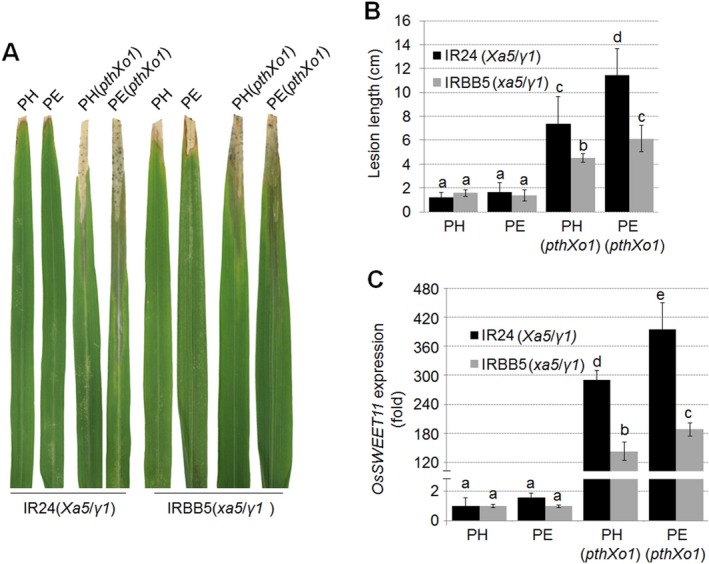
Effects of *OsTFIIAγ1* on PthXo1‐induced lesion length and PthXo1‐activated *OsSWEET11* expression. Disease phenotypes (A) and lesion lengths (B) in IR24 and IRBB5 rice inoculated with *Xanthomonas oryzae* pv. *oryzae* (*Xoo*) PH, PE, PH(*pthXo1*) and PE(*pthXo1*). Five rice leaves were inoculated by tip‐cutting; lesions were measured at 14 days post‐inoculation (dpi). One representative lesion of five is shown in (A). The mean values ± standard deviation (SD) (*n* = 5) are shown in (B). (C) *OsSWEET11* expression in IR24 and IRBB5 inoculated with *Xoo* PH, PE, PH(*pthXo1*) and PE(*pthXo1*). The expression of *OsSWEET11* was evaluated by quantitative real‐time reverse transcription‐polymerase chain reaction (qRT‐PCR) at 24h post‐infiltration (hpi). The mean values ± SD (*n* = 3) are shown. Experiments were repeated three times with similar results and a representative result is shown. Significant differences were detected using Student's *t*‐test at *P* < 0.05.

*Xoo* PH, PE, PH(*pthXo1*) and PE(*pthXo1*) were used to inoculate IR24 and IRBB5 rice. Lesion lengths in IR24 inoculated with PH(*pthXo1*) and PE(*pthXo1*) were significantly longer than those induced in IRBB5 rice (Fig. 2A,B). *Xoo* PE(*pthXo1*), which encodes endogenous *pthXo7* combined with introduced *pthXo1*, resulted in more severe BB lesions than PH(*pthXo1*) in both IR24 and IRBB5 rice (Fig. 2A,B). Furthermore, the expression of the *S* gene *OsSWEET11*, which encodes a sucrose transporter targeted by PthXo1, was significantly higher in IR24 than IRBB5 (*xa5* rice) (Fig. 2C). *Xoo* PE(*pthXo1*) induced higher levels of *OsSWEET11* than *Xoo* PH(*pthXo1*) in IR24 and IRBB5 (Fig. 2C). These findings suggest that Xa5 in IR24 rice may foster PthXo1‐activated expression of the *S* gene *OsSWEET11*, which is attenuated by xa5 in IRBB5. In summary, we propose that the activation of OsTFIIAγ1 by PthXo7, which is suggested by Fig. S2C, leads to enhanced expression of *OsSWEET11*.

**Figure 3:**
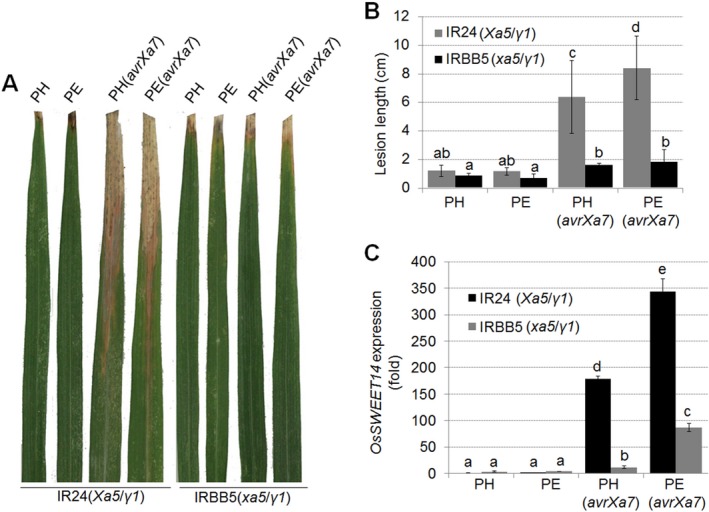
Effect of *OsTFIIAγ1* on AvrXa7‐induced lesion length and AvrXa7‐activated *OsSWEET14* expression. Disease phenotypes (A) and lesion length (B) in rice lines inoculated with *Xanthomonas oryzae* pv. *oryzae* (*Xoo*) PH, PE, PH(*avrXa7*) and PE(*avrXa7*). Five leaves were inoculated; lesions were measured at 14 days post‐inoculation (dpi). One representative lesion of five is shown in (A). The mean values ± standard deviation (SD) (*n* = 5) are shown in (B). (C) *OsSWEET14* expression in IR24 and IRBB5 inoculated with *Xoo* PH, PE, PH(*avrXa7*) and PE(*avrXa7*). The expression of *OsSWEET14* was evaluated by quantitative real‐time reverse transcription‐polymerase chain reaction (qRT‐PCR) at 24h post‐infiltration (hpi). The mean values ± SD (*n* = 3) are shown. Experiments were repeated three times with similar results and one representative result is shown. Significant differences were identified using Student's *t*‐test at *P* < 0.05.

To further investigate the interplay between OsTFIIAγ1, TALEs and *R*/*S* gene targets, we introduced *avrXa7* into *Xoo* PE and PH (Fig. S3; Table S1). The TALE AvrXa7 is a major virulence factor in *Xoo* that activates the expression of *OsSWEET14*, another known *S* gene in rice (Antony *etal*., 2010). *Xoo* PE and PH strains containing *avrXa7* were inoculated to IR24 and IRBB5 rice, and lesions were observed at 14 dpi. BB lesions in IR24 rice inoculated with *Xoo* PH(*avrXa7*) were 6cm in length and dramatically shorter than those caused by PE(*avrXa7*); however, the lesion lengths in IRBB5 rice were less than 2cm (Fig. 3A,B). *OsSWEET14* expression in rice was correlated with lesion length, e.g. higher levels of *OsSWEET14* transcription were observed in IR24 rice inoculated with *Xoo* PE(*avrXa7*) than PH(*avrXa7*) (Fig. 3C). We also noticed significantly higher expression of *OsSWEET14* in IRBB5 rice inoculated with *Xoo* PE(*avrXa7*) than PH(*avrXa7*) (Fig. 3C), suggesting that the endogenous copy of *pthXo7* in *Xoo* PE may contribute to the enhanced *OsSWEET14* expression that is activated by AvrXa7, perhaps via *OsTFIIAγ1*.

**Figure 4:**
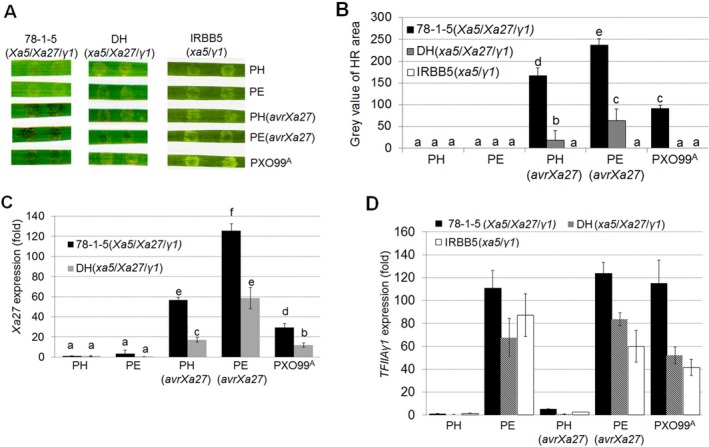
Effect of *OsTFIIAγ1* on *Xa27*‐mediated resistance and *Xa27* expression. (A) Phenotypes in rice lines 78‐1‐5, DH and IRBB5 infiltrated with *Xanthomonas oryzae* pv. *oryzae* (*Xoo*) strains PH, PE, PH(*avrXa27*), PE(*avrXa27*) and PXO99^A^. Each bacterial strain was infiltrated into three leaves with inoculated areas per leaf. One representative photograph was taken at 4 days post‐inoculation (dpi). (B) Quantification of the hypersensitive response (HR) in (A). Values represent the mean size of the grey (necrotic) regions ± standard deviation (SD) (*n* = 3). (C) *Xa27* expression in rice lines 78‐1‐5 and DH inoculated with *Xoo* strains PH, PE, PH(*avrXa27*), PE(*avrXa27*) and PXO99^A^. The expression of *Xa27* was evaluated by quantitative real‐time reverse transcription‐polymerase chain reaction (qRT‐PCR) at 24h post‐infiltration (hpi). (D) Os*TFIIAγ1* expression levels in rice lines 78‐1‐5, DH and IRBB5 infiltrated with *Xoo* strains PH, PE, PH(*avrXa27*), PE(*avrXa27*) and PXO99^A^. The expression of Os*TFIIAγ1* was evaluated by qRT‐PCR at 24 hpi. Values in (C) and (D) represent the mean ± SD (*n* = 3). Experiments were repeated three times with similar results, and one representative result is shown. Significant differences were detected using Student's *t*‐test at *P* < 0.05.

We also investigated how an executor *R* gene contributes to BB resistance when *OsTFIIAγ1* is induced. For these experiments, we transferred *avrXa27*, which is the activator of *Xa27* (Gu *etal*., 2005), into PH and PE strains (Fig. S3). *Xoo* strains PE, PH, PE(*avrXa27*) and PH(*avrXa27*) were then infiltrated into rice using needleless syringes. The three rice lines selected were 78‐1‐5 (containing *Xa27, Xa5* and *OsTFIIAγ1*) (Hu *etal*., 2007), DH (*Xa27, xa5* and *OsTFIIAγ1*) (Gu *etal*., 2009) and IRBB5 (*xa5* and *OsTFIIAγ1*). *Xoo* strains containing *avrXa27*, e.g. PH(*avrXa27*), PE(*avrXa27*) and PXO99^A^, triggered a typical hypersensitive response (HR) in 78‐1‐5 rice; however, IRBB5 rice exhibited a water‐soaked, compatible interaction in response to all strains (Fig. 4A). Surprisingly, the HR in DH rice (*xa5*/*Xa27*/*γ1*) was not as robust as in 78‐1‐5 rice (*Xa5*/*Xa27*/*γ1*), although PE(*avrXa27*) did promote an obvious HR in DH rice (Fig. 4A,B). The findings suggest potential interplay between *Xa5* and *OsTFIIAγ1* in 78‐1‐5 rice that fosters resistance and promotes HR that is mediated by the AvrXa27–XA27 interaction.

To build on these observations, we evaluated *Xa27* expression in 78‐1‐5 and DH rice lines at 24h after infiltration with these bacterial strains. qRT‐PCR indicated that *Xa27* expression was two‐ to three‐fold higher in 78‐1‐5 rice (*Xa5*/*Xa27*/*γ1*) than DH rice (*xa5*/*Xa27*/*γ1*) when inoculated with PH(*avrXa27*), PE(*avrXa27*) and PXO99^A^ (Fig. 4B). The highest *Xa27* expression levels were observed in 78‐1‐5 rice inoculated with PE(*avrXa27*) (Fig. 4B). These results suggest that *Xa5* and *OsTFIIAγ1* in 78‐1‐5 rice promote XA27‐mediated resistance; however, this resistance is attenuated in DH rice, potentially as a result of the absence of *Xa5*. We also observed elevated *OsTFIIAγ1* expression in all three rice lines inoculated with *Xoo* PE, PE(*avrXa27*) and PXO99^A^ (Fig. 4C); these three strains all encode a functional copy of *pthXo7*, a known activator of *OsTFIIAγ1* expression.

The results presented above (Figs 2, 3, 4) lead us to speculate that *OsTFIIAγ1* may partially compensate for the attenuated response to TALEs in *xa5* rice (e.g. IRBB5 and DH). This hypothesis is based on several observations. First, *Xoo* PE, which contains an endogenous copy of *pthXo7*, activates *OsTFIIAγ1* expression in IRBB5 (*xa5*) rice (Fig. S2C). Second, *Xoo* PE containing the three introduced TALEs (*pthXo1*, *avrXa7* and *avrXa27*) induces higher levels of target gene expression (e.g *OsSWEET11*, *OsSWEET14* and *Xa27*) than *Xoo* PH (Figs 2, 3, 4). Finally, in *xa5* rice (IRBB5, DH), TALE target gene expression is lower than in *Xa5* lines (IR24, 78‐1‐5) (Figs 2, 3, 4). However, we observed a modest increase in target gene expression in IRBB5 and DH rice inoculated with *Xoo* strains containing endogenous *pthXo7*, and this was correlated with an increase in *OsTFIIAγ1* expression. Based on these observations, our next experiments were designed to determine whether *OsTFIIAγ1* compensates for *xa5*, potentially by interacting with individual TALEs.

### *OsTFIIAγ1‐*inactive rice plants are more resistant to BB

To directly test the hypothesis that *OsTFIIAγ1* can compensate for the absence of *Xa5*, we constructed rice lines containing defective forms of *OsTFIIAγ1*. This was accomplished by editing *OsTFIIAγ1* in IRBB5 using Clustered regularly interspaced short palin dromic repeats and CRISPR‐associated protein 9 (CRISPR/Cas9) technology (Zhou *etal*., 2014). Although the sequences of *xa5* and *OsTFIIAγ1* are similar, we designed a single‐guide RNA (sgRNA) sequence that specifically binds *OsTFIIAγ1* (Fig. S4A,B, see Supporting Information). We generated 12 edited rice lines, and sequence analysis showed that they were genetically modified and homozygous (Fig. S4C). Two of these rice lines, designated *TF1–2* and *TF1–5*, both had single nucleotide insertions (Fig. S4D) in *OsTFIIAγ1* and were used in further studies.

To confirm that *OsTFIIAγ1* was defective and not expressed as a functional protein in the *TF1–2* and *TF1–5* rice lines, the expression of the protein products was investigated. For these experiments, *OsTFIIAγ1* and its defective derivatives, *TF*‐2 and *TF*‐5, were cloned in a yellow fluorescent protein (YFP) expression vector in which only functional proteins generate the fluorescence signal (Table S1). These YFP constructs were transiently expressed in *Nicotiana benthamiana* as described in Methods S3 (see Supporting Information). The OsTFIIAγ1::YFP fusion was clearly localized to the plasma membrane and nuclei; however, YFP was not expressed in tobacco transformed with TF‐2::YFP or TF‐5::YFP, which indicates that these modified forms of *OsTFIIAγ1* were not expressed as functional proteins (Fig. S4E).

**Figure 5:**
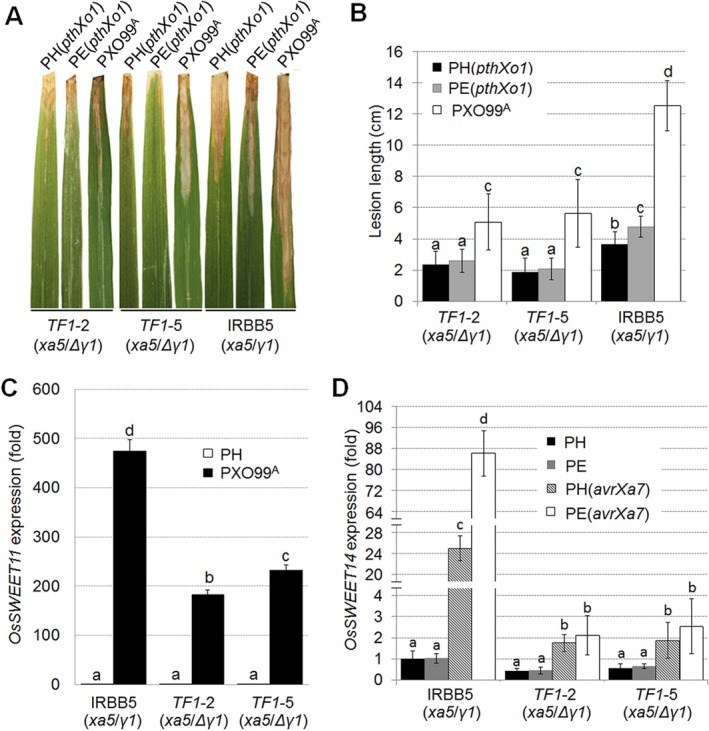
Disease phenotypes and *S* gene levels in IRBB5 (wild‐type *OsTFIIAγ1*), *TF1*‐2 and *TF1*‐5 rice (defective *OsTFIIAγ1*). Disease phenotypes (A) and lesion lengths (B) in *TF1*‐2, *TF1*‐5 and IRBB5 rice at 14 days post‐inoculation (dpi) with *Xanthomonas oryzae* pv. *oryzae* (*Xoo*) PH(*pthXo1*), PE(*pthXo1*) and PXO99^A^. A typical lesion (*n* = 4) is shown in (A); mean values ± standard deviation (SD) (*n* = 4) are shown in (B). (C) *OsSWEET11* expression levels in IRBB5, *TF1–2* and *TF1–5* rice at 24h post‐infiltration (hpi) with *Xoo* PH and PXO99^A^. (D) *OsSWEET14* expression in IRBB5, *TF1–2* and *TF1–5* rice inoculated with *Xoo* PH, PE, PH(*avrXa7*) and PE(*avrXa7*). Abbreviations: *γ1*, wild‐type *OsTFIIAγ1*; Δ*γ1*, mutated *OsTFIIAγ1*. Values in (C) and (D) represent the mean ± SD (*n* = 3), and significant differences were detected using Student's *t*‐test at *P* < 0.05. One representative result of three biological replicates is shown.

The impact of defective *OsTFIIAγ1* on *Xoo*–rice interactions was investigated by inoculating PXO99^A^, PH(*pthXo1*) and PE(*pthXo1*) strains to *TF1* rice lines and IRBB5 using the tip‐cutting method. Lesions in *TF1* lines inoculated with wild‐type PXO99^A^ were significantly smaller than those in IRBB5 rice (Fig. 5A,B). Interestingly, both PH(*pthXo1*) and PE(*pthXo1*) strains induced more severe symptoms in IRBB5 than in *TF1* lines (Fig. 5A,B). No obvious differences in disease symptoms or lesion lengths were observed in *TF1* lines inoculated with PH(*pthXo1*) or PE(*pthXo1*) strains.

Our results (Figs 2, 3, 4) suggest a complex interplay between *OsTFIIAγ1* and the rice *S* genes encoded by *OsSWEET11* and *OsSWEET14*. Thus, we examined the expression of these *S* genes in IRBB5 and *TF1* rice inoculated with *Xoo* PXO99^A^, PH(*avrXa7*) and PE(*avrXa7*). *Xoo* PXO99^A^ contains an endogenous copy of *pthXo1*, which activates *OsSWEET11*. When *Xoo* PXO99^A^ was used as inoculum, the expression of *OsSWEET11* was significantly lower in *TF1* lines relative to IRBB5 rice (Fig. 5C). Thus, the defective *OsTFIIAγ1* in *TF1* lines had a direct, negative impact on expression of the *S* gene, *OsSWEET11*, and this was correlated with reduced virulence (Fig. 5A,B). We also evaluated *OsSWEET14* expression in IRBB5 and *TF1* rice inoculated with PH and PE containing *avrXa7*, which specifically activates this *S* gene. *OsSWEET14* expression was significantly lower in *TF1* lines inoculated with PH(*avrXa7*) and PE(*avrXa7*) than in IRBB5 rice (Fig. 5D). This is further evidence that the defective copy of *OsTFIIAγ1* compromises the virulence of *Xoo* in the *TF1* lines. Collectively, these results indicate that *OsTFIIAγ1* promotes TALE‐mediated *S* gene transcription, and this function is more apparent in *xa5* rice lines, such as IRBB5.

### *Xoo* GX4 containing *pthXo7 in trans* causes disease in IRBB5 rice

**Figure 6:**
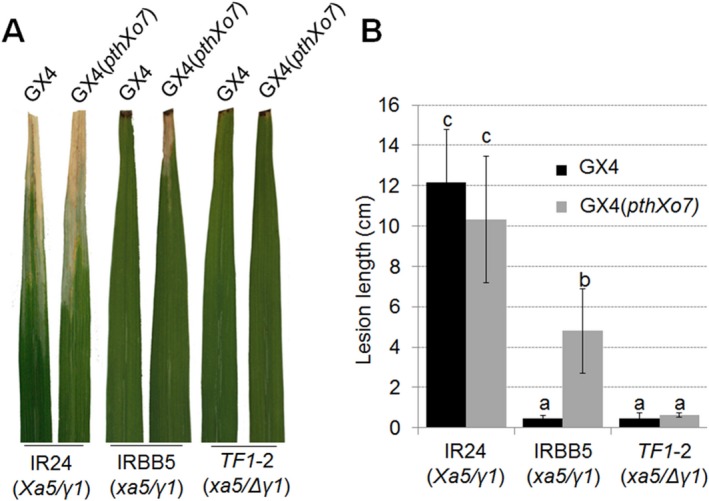
Disease symptoms in IR24, IRBB5 and *TF1* rice lines inoculated with *Xanthomonas oryzae* pv. *oryzae* (*Xoo*) GX4 and GX4(*pthXo7*). Symptoms (A) and lesion lengths (B) at 14 days post‐inoculation (dpi) are shown. One representative leaf (*n* = 4) is shown in (A). Mean values ± standard deviation (SD) (*n* = 4) are shown in (B). One representative result from three biological repeats with similar results is shown. Abbreviations: *γ1*, *OsTFIIAγ1*; Δ*γ1*, mutated *OsTFIIAγ1*.

*OsTFIIAγ1* is activated by PthXo7 (Sugio *etal*., 2007) and can compensate for the absence of *Xa5* in IRBB5 rice (Fig. 5C); thus, we speculated that the expression of *pthXo7* in a *Xoo* strain lacking this gene might result in disease when inoculated to IRBB5 rice. *Xoo* GX4 was chosen for these experiments; this strain was non‐pathogenic when inoculated to IRBB5 rice (Fig. 1A) and did not induce *OsTFIIAγ1* expression (Fig. 1B). *Xoo* GX4 was transformed with pHZWpthXo7 and the overproduction of PthXo7 was verified by immunoblotting (Fig. S3B). *Xoo* GX4 and GX4(*pthXo7*) were then inoculated to IR24 (*Xa5*/*γ1*), IRBB5 (*xa5*/*γ1*) and *TF1*‐2 (*xa5*/Δ*γ1*). There was no obvious difference in lesion length or symptoms in IR24 rice inoculated with *Xoo* GX4 or GX4(*pthXo7*) (Fig. 6). Wild‐type *Xoo* GX4 did not cause disease in IRBB5 rice; however, *Xoo* GX4(*pthXo7*) gained the ability to induce small lesions (∼4.5cm) in IRBB5 (Fig. 6A,B). Intriguingly, both *Xoo* GX4 and GX4(*pthXo7*) were non‐pathogenic in *TF1* rice (Fig. 6A,B), which lacks a functional copy of *OsTFIIAγ1*. These results suggest that the activation of OsTFIIAγ1 by PthXo7 contributes to lesion development in the IRBB5/GX4(*pthXo7*) interaction; we also speculate that *OsTFIIAγ1* partially compensates for the lack of *Xa5* in IRBB5 rice.

### TALEs interact with OsTFIIAγ, Xa5 and xa5 with different affinities

Bioinformatics analysis of OsTFIIAγ1, Xa5 and xa5 indicated that OsTFIIAγ1 shares the 39th valine residue with Xa5, but not with xa5 (Fig. S4A). Given that Xa5 interacts with several characterized TALEs (Yuan *etal*., 2016) and is highly similar to OsTFIIAγ1, we speculate that OsTFIIAγ1 also associates with a variety of TALEs. To address this hypothesis, we investigated the direct interaction of OsTFIIAγ1, Xa5 and xa5 with PthXo1, AvrXa7 and AvrXa27 using bimolecular fluorescence complementation (BiFC). For these experiments, OsTFIIAγ1, Xa5 and xa5 were fused with YN (N‐terminus of YFP), and PthXo1, AvrXa7 and AvrXa27 were fused with YC (C‐terminus of YFP), as detailed in Methods S3 and Table S1. *Agrobacterium*‐mediated transformation was used to introduce these constructs into *N. benthamiana* for transient expression and BiFC.

**Figure 7:**
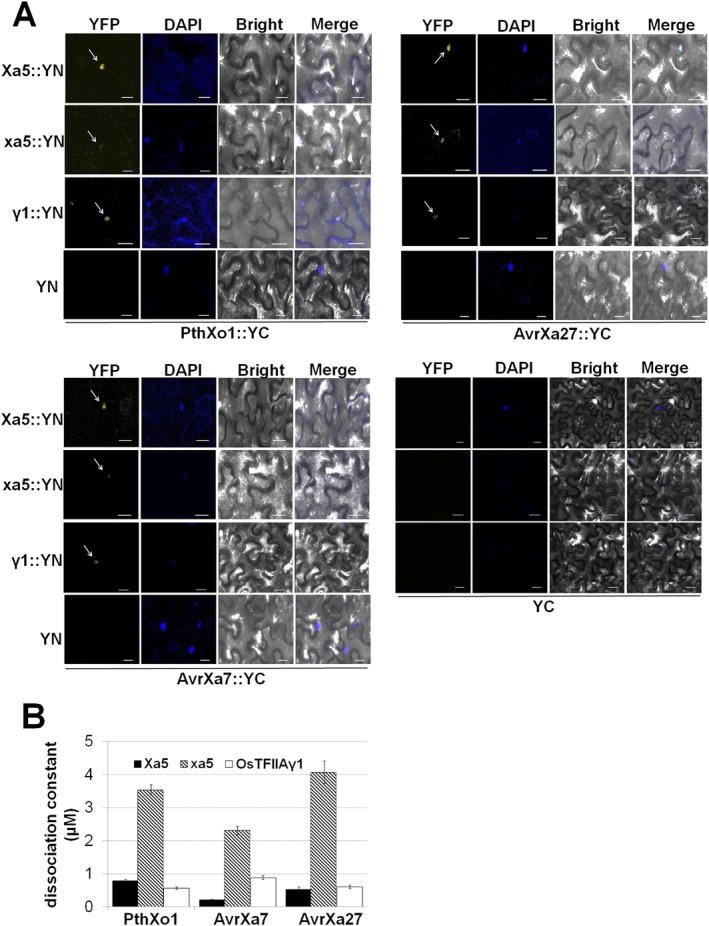
Interaction of Xa5, xa5 and OsTFIIAγ1 with PthXo1, AvrXa27 and AvrXa7 using biomolecular fluorescence complementation (BiFC) and microscale thermophoresis (MST). (A) BiFC visualization of the interaction between YN‐tagged TALEs and YC‐tagged OsTFIIAγ subunits in tobacco leaves. The fluorescence in the yellow fluorescent protein (YFP) panels occurred when YN‐labelled TALES interacted with YC‐labelled OsTFIIAγ subunits (see arrows). Controls included *Nicotiana benthamiana* transformed with empty YN vector and YC‐tagged TALEs, and empty YC vector and YN‐tagged OsTFIIAγ. Nuclei were stained by 4′,6‐diamidino‐2‐phenylindole (DAPI). Bars represent 20 μm. (B) Binding affinity of TALEs (PthXo1, AvrXa7 and AvrXa27) and labelled Xa5, xa5 and OsTFIIAγ1 as measured by MST. OsTFIIAγ subunits were labelled with the amine‐reactive, red fluorescent dye NT‐647 and mixed with 16 different concentrations of purified TALEs (Fig. S5, see Supporting Information). The affinity of the nine interactions is represented by the dissociation constant (*K*_d_). Values represent the means ± standard deviation (SD) (*n* = 3). Experiments were repeated twice with similar results.

The co‐expression of Xa5::YN with the three YC‐tagged TALEs resulted in a fluorescent signal, indicating that PthXo1::YC, AvrXa27::YC and AvrXa7::YC form a complex with Xa5 in plant nuclei (Fig. 7A, see arrows). Similarly, the co‐expression of OsTFIIAγ1::YN (γ1::YN) with PthXo1::YC, AvrXa27::YC or AvrXa7::YC also resulted in fluorescent plant nuclei (Fig. 7A), which further supports the interaction of OsTFIIAγ1 with TALEs. However, the co‐expression of xa5::YN with PthXo1::YC, AvrXa27::YC or AvrXa7::YC resulted in weaker fluorescence relative to Xa5::YN and OsTFIIAγ1::YN (Fig. 7A). These results suggest that xa5 also associates with TALEs, but the affinity is much lower than that observed for Xa5 and OsTFIIAγ1.

To gain more information about the binding affinities of Xa5, xa5 and OsTFIIAγ1 and the three TALEs (PthXo1, AvrXa7 and AvrXa27), we used microscale thermophoresis (MST). Sixteen different concentrations of the purified TALE proteins (PthXo1, AvrXa7 and AvrXa27) were mixed with labelled OsTFIIAγ1, Xa5 and xa5, and subjected to MST (Fig. S5, see Supporting Information). When Xa5 and OsTFIIAγ1 were combined with PthXo1, AvrXa7 or AvrXa27, the *K*_d_ values were relatively small (less than 1 μm; Fig. 7B), indicating strong affinity for the TALEs. Although xa5 interacted with the three TALES, the *K*_d_ values were much higher (2.3–4.0 μm), indicating reduced affinity for the TALEs relative to Xa5 and OsTFIIAγ1 (Fig. 7B). Taken together, these results indicate that OsTFIIAγ1 and Xa5 strongly interact with PthXo1, AvrXa7 and AvrXa27 *in vitro*. Conversely, the interaction of these TALEs and xa5 is much weaker than observed with Xa5 and OsTFIIAγ1.

## Discussion

A prerequisite for Pol II‐dependent transcription in eukaryotes is the recruitment of general transcription factors, e.g. TFIIA, TFIIB, TFIID, TFIIE, TFIIF and TFIIH, to the core promoter region of the target gene. This process begins with the recruitment of TFIIA and TFIID (Buratowski *etal*., 1989; Thomas and Chiang, 2006). TFIIA generally serves as a bridge between the TATA‐box binding protein and lobe B of TFIID, which facilitates TFIID binding to the TATA‐box (Louder *etal*., 2016). In Arabidopsis, TFIIA is composed of two subunits: the large subunit TFIIAαβ and the small subunit TFIIAγ (Li *etal*., 1999). Yuan *etal*. (2016) have recently demonstrated a role for TFIIAγ5 (OsTFIIAγ5, Xa5) in the *Xoo*–rice interaction. In their model, TALEs secreted by *Xoo* interact with Xa5 to facilitate activation of host susceptibility genes. It is also important to mention that *xa5*, a naturally occurring mutant allele of *Xa5*, confers a level of resistance to *Xoo*, which is presumably due to the reduced interaction between TALEs and the Pol II initiation complex when xa5 is present (Schornack *etal*., 2006, 2013).

In the current study, we examined the role of OsTFIIAγ1 in TALE‐mediated interactions. Sugio *etal*. (2007) have previously demonstrated that the TALE PthXo7 activates the expression of *OsTFIIAγ1*, which suggests a complex interplay between multiple transcriptional factors and TALEs, which can foster or impede the transcription of target *R/S* genes. In this study, we showed that the activation of OsTFIIAγ1 increased the TALE‐induced expression of target genes, especially in *xa5*‐containing rice (Figs 2C, 3C and 4C); thus, OsTFIIAγ1 plays a compensatory role in the absence of Xa5. It is important to note that the basal level of *OsTFIIAγ1* expression is much lower than that of *Xa5* in both seedlings and adult rice plants, regardless of pathogen infection (Figs 1D and S1). This observation is consistent with previous research (Iyer and McCouch, 2004) and supports the assumption that *Xoo* evolved or recruited PthXo7 to increase the transcription of *OsTFIIAγ1*, which can then promote TALE‐targeted *R/S* gene expression when Xa5 is mutated to xa5. Furthermore, our results showed that *Xoo* PE, which encodes *pthXo7*, induced a higher expression of target genes than *Xoo* PH when both strains contained the same set of introduced TALEs (Figs 2C and 3C). Taken together, these findings support the contention that the increased expression of *OsTFIIAγ1* via PthXo7 can partially compensate for the attenuated expression of TALE‐targeted *R/S* genes in *xa5* rice.

TALEs bind the EBEs of target genes near the TATA‐box, which generally activates transcription (Grau *etal*., 2013). Our findings indicate that TALEs interact with TFIIAγ subunits (Fig. 7) and form a complex with specific plant transcription factors. These TALE‐containing transcriptional complexes presumably promote target gene expression *in planta*. Recently, Yuan *etal*. (2016) used a yeast two‐hybrid system, and reported that 15 tested TALEs isolated from *Xoo* PXO99^A^ interact with Xa5, but only PthXo1, Tal7a and Tal8a of the 15 TALEs interact with xa5. Interestingly, OsTFIIAγ1 did not interact with full‐length or truncated PthXo1 or the TFB site of 14 other Xoo TALEs (Yuan *etal*., 2016). As a result of the existence of a full set of transcription factors (Poss *etal*., 2013) and the self‐activating ability of TALEs in yeast, the yeast two‐hybrid system may not be the most robust system to test interactions between full‐length TALEs and OsTFIIAγ proteins. Thus, we used BiFC and MST assays to detect interactions *in planta* and *in vitro*, respectively. Both BiFC and MST indicated that three TALEs (PthXo1, AvrXa7 and AvrXa27) interacted with Xa5, xa5 and OsTFIIAγ1; however, the affinities of the three TALEs were significantly higher with Xa5 and OsTFIIAγ1 than with xa5 (Figs 7 and S5).

**Figure 8:**
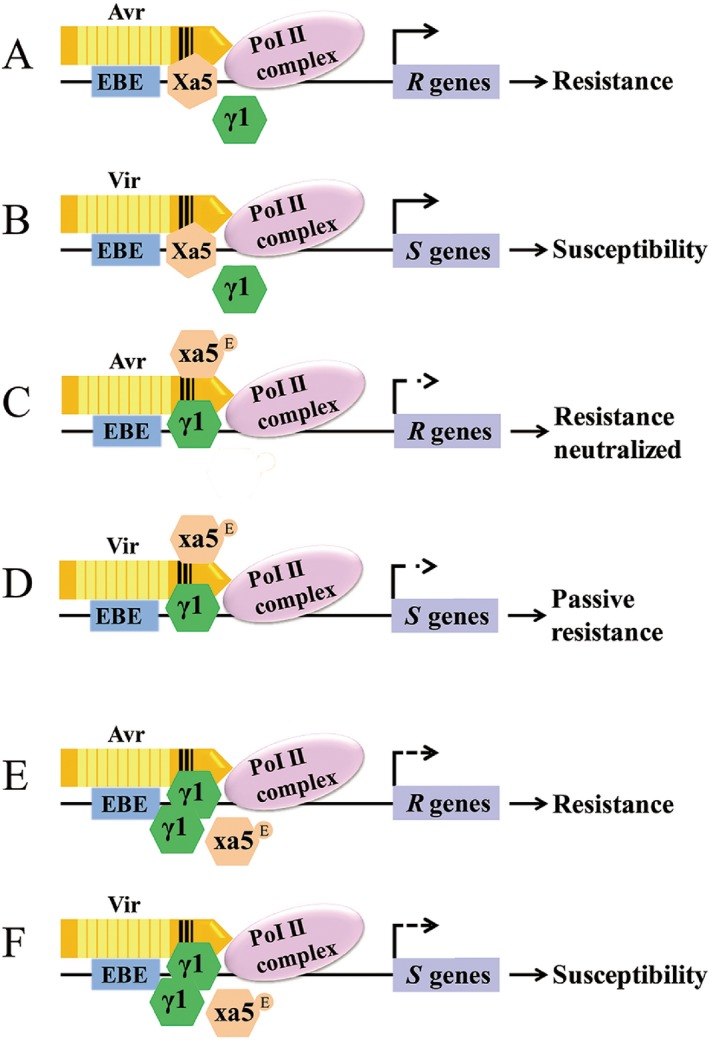
Theoretical model showing how OsTFIIAγ subunits (Xa5, OsTFIIAγ1 and xa5) modulate transcription activator‐like effector (TALE)‐activated host gene transcription and disease development. (A, B) Avirulent (Avr) and virulent (Vir) TALEs associate with Xa5 to form a transcription factor complex for the initiation of the expression of *R* (A) or *S* (B) genes. Through interaction with TALEs, the low expression level of OsTFIIAγ1 makes it play a minor role in the activation of *R* or *S* genes. (C, D) Rice lines are homozygous for xa5. The weaker affinity of the xa5–TALE association facilitates OsTFIIAγ1 binding (green hexagons); however, the relatively low level of OsTFIIAγ1 prevents TALE‐mediated *R* or *S* gene activation and leads to neutralized (C) or passive (D) resistance. (E, F) The elevated copy number of OsTFIIAγ1 when transcription is enhanced by PthXo7 (not shown). In this scenario, OsTFIIAγ1 plays a compensatory role for Xa5 in the *xa5* background, and this leads to *R* or *S* gene expression and an enhanced level of resistance (E) or susceptibility (F). Arrows with a single dash ( 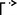 ) indicate transcriptional inhibition and failure to express the target *R* or *S* gene. Arrows with multiple dashes ( 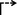 ) indicate elevated transcription, which results in an enhanced level of resistance or susceptibility. Abbreviations: Avr, avirulent TALE; EBE, effector‐binding element; Pol II, polymerase II; Vir, virulent TALE; γ1, OsTFIIAγ1.

To better describe our observations and the potential roles of Xa5, xa5 and OsTFIIAγ1 in the activation of TALE‐targeted (*R* or *S*) genes, we present a model based on previous reports and the findings in our study (Fig. 8). In *Xa5* rice, the expression of *Xa5* is much higher than *OsTFIIAγ1*, which enables TALEs to function as transcription binding proteins (TBPs) in avirulent (Avr) or virulent (Vir) forms. The TALEs form a transcription complex together with Xa5 and other rice transcription factors, and this activates *R* or *S* gene expression, leading to disease resistance (Fig. 8A) or susceptibility (Fig. 8B). However, in *xa5* rice, the reduced association of TALEs with xa5 results in a less effective transcription complex, leading to the suppression of TALE‐targeted gene expression. In this scenario, the BB resistance mediated by avirulent TALEs is neutralized (Fig. 8C) or the susceptibility mediated by virulent TALEs is suppressed and results in passive resistance (Fig. 8D), which is consistent with previous reports (Gu *etal*., 2009; Huang *etal*., 2016). An exception to this part of the model is the virulence of the PH(*pthXo1*) strain, which causes BB in *xa5* rice and *OsTFIIAγ1‐*defective rice lines (*TF1‐2* and *TF1‐5*) (Fig. 5). It should be noted that the PH(*pthXo1*) lesions are much smaller than those caused by PE(*pthXo1*) (Fig. 5A,B). The reason for this may be the weak affinity of xa5 with PthXo1 (Fig. 7), which is consistent with the findings reported by Yuan *etal*. (2016), who showed that xa5 interacts with PthXo1. Thus, PthXo1‐containing *Xoo* strains retain virulence and are compatible with *xa5* rice.

To overcome xa5‐mediated resistance, *Xoo* strains, such as PXO99^A^, use PthXo7 to increase the transcription of *OsTFIIAγ1* (Fig. 1C). Furthermore, it is important to mention that the affinities of OsTFIIAγ1 and Xa5 with the three tested TALE proteins were similar and much higher than the affinity of TALEs for xa5 (Fig. 7). Thus, we speculate that the binding of OsTFIIAγ1 can cause the formation of a transcriptional complex that facilitates TALE‐activated target gene expression. It is important to consider the elevated copy number of OsTFIIAγ1 that occurs when transcription is enhanced by PthXo7. In this scenario, OsTFIIAγ1 can play a compensatory role for Xa5 in the *xa5* background, and this leads to *R* or *S* gene expression and some level of resistance (Fig. 8E) or susceptibility (Fig. 8F). In some cases, an *R* rice line may show elevated resistance to the pathogen carrying the cognate avirulent TALE (Fig. 8E). Conversely, an *S* rice line may show enhanced susceptibility to *Xoo* strains harbouring the associated virulent TALE (Fig. 8F). An example of the complex interplay between transcription factors can also be observed with *Xoo* GX4, which activates transcription of *OsSWEET14* (Fig. S6, see Supporting Information) and is avirulent (incompatible) in *xa5* rice (Fig. 6). However, *Xoo* GX4(*pthXo7*) was able to induce some disease symptoms in *xa5* rice. Future studies are underway to clarify the association of TALEs with Xa5, xa5 andOsTFIIAγ1, and how TALEs specifically activate *R* or *S* gene expression.

In this study, we also generated an inactive form of *OsTFIIAγ1* in *xa5* rice using CRISPR/Cas9 technology (Fig. 4). The genetically modified *TF1* rice lines retained resistance to GX4 (lacks *pthXo7*) and enhanced resistance to PXO99^A^ and GX4(*pthXo7*) (Figs 5 and 6). These results suggest that *TF1* lines will be valuable in future efforts to evade *Xoo* and reduce BB symptoms in rice breeding programmes.

## Experimental procedures

### Bacterial strains, plasmids and plant materials

The bacterial strains and plasmids used in this study are listed in Table S1. *Escherichia coli* strains were cultivated in Luria–Bertani medium at 37°C (Chong, 2001). *Xanthomonas* strains were cultured in nutrient broth (NB) or NB amended with agar at 28°C (Li *etal*., 2011). *Agrobacterium* was cultured in Luria–Bertani medium containing rifampicin at 28°C. Antibiotics were used at the following final concentrations: ampicillin, 100 µg/mL; rifampicin, 75 µg/mL; kanamycin, 25 µg/mL; spectinomycin, 50 µg/mL.

Indica rice IRBB5 (harbouring *xa5*) and IR24 were obtained from the International Rice Research Institute. DH, the rice line containing the two homozygous resistance genes *Xa27* and *xa5*, was kindly provided by Zhongchao Yin (Gu *etal*., 2009). Rice line 78‐1‐5, containing *Xa27*, was obtained from Chaozu He (Hu *etal*., 2007). All rice plants were grown at 28°C in a glasshouse at Shanghai Jiao Tong University with a 12‐h photoperiod.

### Plant infection and HR assays

HR assays were carried out as described previously (Hopkins *etal*., 1992). Briefly, three to five leaves of 3‐week‐old rice plants were infiltrated with bacterial suspensions [optical density at 600nm (OD_600_) = 0.6] using a needleless syringe. The quantification of HR was analysed by measuring the grey, necrotic regions in leaf tissue using Fiji software (Schindelin *etal*., 2012; Sekulska‐Nalewajko *etal*., 2016). For measurement of lesion lengths, three to five leaves from 6–8‐week‐old rice plants were inoculated with bacterial suspensions (OD_600_ = 0.6) using a tip‐cutting method (Kauffman *etal*., 1973). Both disease and HR assays were performed at least three times, and Student's *t*‐test was used for significance (*P* < 0.05).

### RNA extraction and gene expression analysis

At 24h post‐infiltration (hpi), leaves of inoculated rice seedlings were selected and frozen in liquid nitrogen. For RNA extraction, frozen samples were pulverized, suspended in 1ml of RNAiso Plus (Takara, Dalian, China) and precipitated with isopropanol. RNA (1 μg) was then added for cDNA synthesis using EasyScript^®^ One Step gDNA Removal and cDNA Synthesis Supermix (TransGen, Beijing, China). Synthesized cDNA (20 μL) was diluted to 100 μL and used for qRT‐PCR employing TransStart^®^ Tip Green qPCR SuperMix (TransGen). qRT‐PCR was performed using an ABI 7500 quantitative PCR system. Fold change in gene expression was measured using the 2^–△△Ct^ method (Livak and Schmittgen, 2001). The primer sequences are provided in Table S2 (see Supporting Information).

### Modification of IRBB5 rice line using the CRISPR/Cas9 system

IRBB5 rice was genetically modified using CRISPR/Cas9 technology as described previously (Zhou *etal*., 2014). Briefly, the sgRNA targeted a 20‐bp region (5′‐GACCATGTCGTCCAGCGTGT‐3′, minus strand) in the first exon of *OsTFIIAγ1*; this sequence was driven by the rice *U6.2* promoter. The sgRNA and Cas9 constructs were transferred into IRBB5 callus cells using *Agrobacterium*‐mediated transformation (Hiei *etal*., 1994), which was a service by Wuhan Biorun Bio‐Tech Co. Ltd., Wuhan, China. Genomic DNA was isolated from leaves of transgenic rice using the Cetyltrimethyl Ammonium Bromide method (Zhou *etal*., 2014). Genomic DNA was employed for PCR amplification of the *OsTFIIAγ1* region using the primer pair TFIIAγ1‐YN‐F(*Xba*I)/TFIIAγ1‐test‐R (Table S2). The resulting amplicons were cloned into the pMD18‐T vector (Takara) using the TA cloning method; clones with confirmed inserts were then sequenced.

### BiFC experiments

For BiFC experiments, *Xa5*, *xa5* and *OsTFIIAγ1* were amplified using the primer pairs Xa5‐YN‐F(*Xba*I)/Xa5‐YN‐R(*Sma*I) and TFIIAγ1‐YN‐F(*Xba*I)/TFIIAγ1‐YN‐R(*Sma*I). *Xa5*, *xa5* and *OsTFIIAγ1* were inserted into the N‐terminus of the YFP (YN) vector using *Xba*I and *Sma*I (see Methods S3), resulting in Xa5::YN, xa5::YN and OsTFIIAγ1::YN, respectively.

BiFC assays were performed as described previously with minor modifications (Walter *etal*., 2004). Briefly, *Agrobacterium* GV3101 strains containing YN and YC constructs were cultured to OD_600_ = 1.5, harvested by centrifugation and resuspended in inducing buffer (10mm MgCl_2_, 0.2mm acetosyringone and 200 mm 2‐(4‐Morpholino) ethanesulfonic acid (MES), pH 5.6) to OD_600_ = 1.0. Buffer‐supplemented *Agrobacterium* strains containing the YN constructs (Xa5::YN, xa5::YN and OsTFIIAγ1::YN) and YC constructs (pthXo1::YC, avrXa7::YC and avrXa27::YC) were mixed in a ratio of 1 : 1 and incubated at 25°C for 1h. Controls included *N. benthamiana* transformed with empty YN vector and YC‐tagged TALE, and empty YC vector and YN‐tagged OsTFIIAγ. The induced *Agrobacterium* mixtures were infiltrated into *N. benthamiana* leaves for transient expression. At 48 hpi, fluorescence was imaged with a confocal laser fluorescence microscope and 4′,6‐diamidino‐2‐phenylindole (DAPI, 100 μg/mL) was used for nuclei staining.

### MST experiments

To assess interactions between TALEs and OsTFIIAγ subunits, MST was performed as described previously (Cai *etal*., 2017; Wienken *etal*., 2010). His‐tagged TALEs and OsTFIIAγ subunits were purified from the pET30a constructs (Methods S4, see Supporting Information) with Ni‐NTA His‐Bind resin. The purified protein buffer was exchanged for MST buffer [50mm Tris‐HCl (pH 7.8) with 150mm NaCl, 10mm MgCl_2_ and 0.05% Tween‐20], and protein concentrations were determined using the Bradford method. OsTFIIAγ proteins were labelled with the amine‐reactive, red fluorescent dye NT‐647 using the Monolith NT.115 Protein Labeling Kit as recommended by the manufacturer (NanoTemper Technologies, Germany), and then eluted with MST buffer. Sixteen different concentrations of TALE proteins starting from 10 μm were made by two‐fold serial dilutions. Different concentrations of TALEs were mixed with 1 μm labelled OsTFIIAγ proteins in a 1 : 1 (v/v) ratio. After a 10‐min incubation at room temperature, the samples were loaded into silica capillaries. Measurements were performed at 25°C using 35% LED power and 80% IR laser power. MST was performed with a Monolith NT.115T (NanoTemper Technologies), and data were analysed using NTAnalysis v. 1.5.41.

## Author contributions

W.M. and G.C. designed the experiments; W.M. performed the experiments; L.Z., Z.J. and X.X. contributed materials; Z.X. provided technical assistance for sgRNA design; Y.Y. isolated *Xoo* strains from China rice fields; W.M. and G.C. wrote the paper; J.R.A. revised the manuscript; all authors read, commented on and approved the manuscript.

## Conflicts of interest

The authors declare no conflicts of interest.

## Supporting information

Additional supporting information may be found in the online version of this article at the publisher's web‐site

**Fig. S1** Basal expression level of *OsTFIIAγ1* and *Xa5/xa5* in IR24 and IRBB5 rice lines as analysed by real‐time= reverse transcription‐polymerase chain reaction (RT‐PCR). *OSActin* was used as an internal control. NTC, no template control.

**Fig. S2** Functional map showing the two *tal*‐deletion mutants [*Xanthomonas oryzae* pv. *oryzae* (*Xoo*) PE and PH] derived from PXO99^A^ and the expression of *OsTFIIAγ1*.

**Fig. S3** Western blot analysis of transcription activator‐like effector (TALE) production in various *Xanthomonas oryzae* pv. *oryzae* (*Xoo*) strains.

**Fig. S4** Modification of *OsTFIIAγ1* in IRBB5 rice by Clustered regularly interspaced short palin dromic repeats and CRISPR‐associated protein 9 editing.

**Fig. S5** The affinity of PthXo1, AvrXa7 and AvrXa27 for Xa5, xa5 and OsTFIIAγ using microscale thermophoresis (MST).

**Fig. S6** *Xanthomonas oryzae* pv. *oryzae* (*Xoo*) strain GX4 induces the expression of *OsSWEET14*, but not *OsSWEET11*, in IR24 rice.

**Methods S1** DNA manipulation and plasmid construction.

**Methods S2** Immunoblotting assays.

**Methods S3** Assembly of yellow fluorescent protein (YFP)‐tagged constructs.

**Methods S4** Protein production and purification.

**Table S1** Strains and plasmids used in this study.

**Table S2** Primers used in this study.
