# Supplementary material for: Xanthomonas oryzae pv. oryzae TALE proteins recruit OsTFIIAγ1 to compensate for the absence of OsTFIIAγ5 in bacterial blight in rice
Source: Mol Plant Pathol. 2018 Aug 7;19(10):2248–62. doi: 10.1111/mpp.12696 (PMC6638009; doi:10.1111/mpp.12696)
Supplement: Supplementary file 1 — Fig. S1 Basal expression level of OsTFIIAγ1 and Xa5/xa5 in IR24 and IRBB5 rice lines as analysed by real‐time= reverse transcription‐polymerase chain reaction (RT‐PCR). OSActin was used as an internal control. NTC, no template control. [file MPP-19-2248-s001.docx]

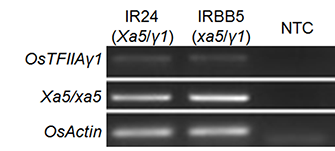


**Figure S1.** Basal expression level of *OsTFIIAγ1* and *Xa5/xa5* in IR24 and IRBB5 rice lines as analyzed by RT-PCR. *OsActin* was used as an internal control. NTC, no template control.
