# Supplementary material for: Xanthomonas oryzae pv. oryzae TALE proteins recruit OsTFIIAγ1 to compensate for the absence of OsTFIIAγ5 in bacterial blight in rice
Source: Mol Plant Pathol. 2018 Aug 7;19(10):2248–62. doi: 10.1111/mpp.12696 (PMC6638009; doi:10.1111/mpp.12696)
Supplement: Supplementary file 2 — Fig. S2 Functional map showing the two tal‐deletion mutants [Xanthomonas oryzae pv. oryzae (Xoo) PE and PH] derived from PXO99A and the expression of OsTFIIAγ1. [file MPP-19-2248-s002.docx]

**
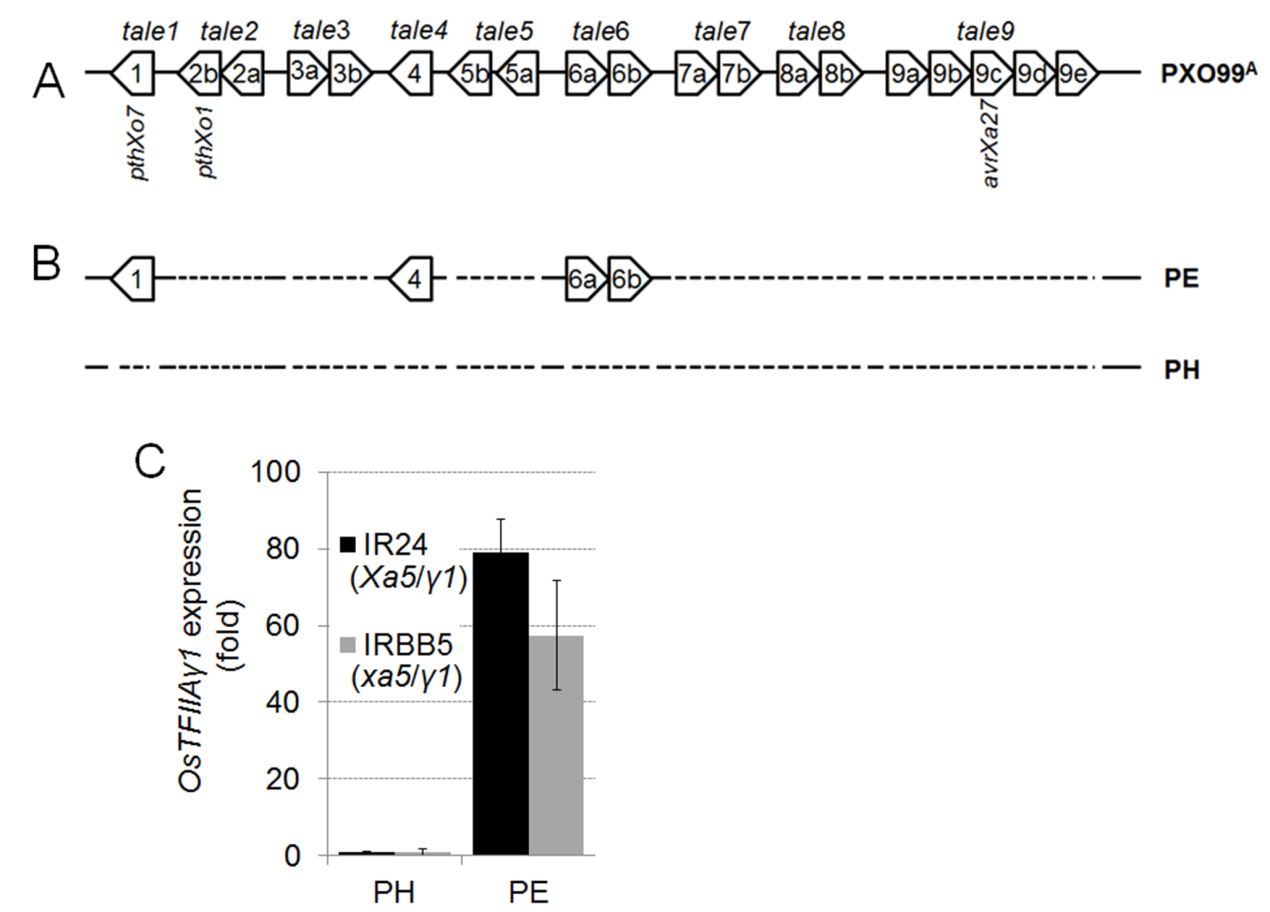
**

**Figure S2.** Functional map showing the two *tal*-deletion mutants (*Xoo* PE and PH) derived from PXO99^A^ and expression of *OsTFIIAγ1*. (A) Functional map of *tal* gene clusters in the PXO99^A^ genome. Open pentagons represent individual *tal* genes. Cluster names are labeled on the top and *tal* genes related to this study are designated with their common names below (e.g. *pthXo7*, *pthXo1*). (B) Function map of *tal* regions in mutant strains *Xoo* PE and PH, which are derived from PXO99^A^. PE contains several functional tal genes, including *pthXo7*; PH is devoid of all *tal* gene clusters. (C) *Xoo* PE but not *Xoo* PH induces *OsTFIIAγ1* expression in IR24 and IRBB5 rice. The expression of *OsTFIIAγ1* was evaluated by qRT-PCR at 24 hpi.
