# Supplementary material for: Xanthomonas oryzae pv. oryzae TALE proteins recruit OsTFIIAγ1 to compensate for the absence of OsTFIIAγ5 in bacterial blight in rice
Source: Mol Plant Pathol. 2018 Aug 7;19(10):2248–62. doi: 10.1111/mpp.12696 (PMC6638009; doi:10.1111/mpp.12696)
Supplement: Supplementary file 3 — Fig. S3 Western blot analysis of transcription activator‐like effector (TALE) production in various Xanthomonas oryzae pv. oryzae (Xoo) strains. [file MPP-19-2248-s003.docx]

**
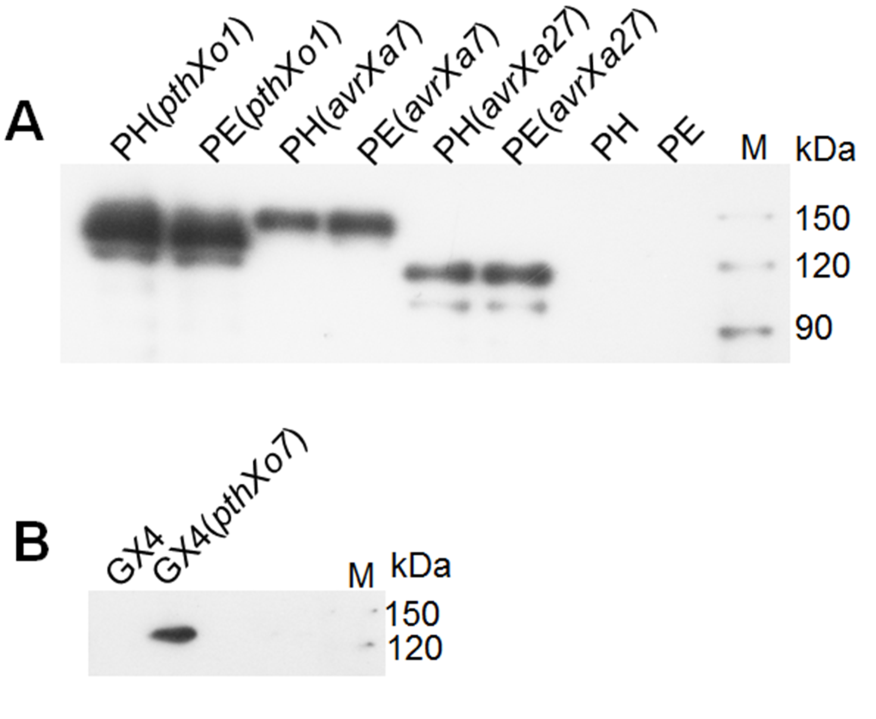
**

**Figure S3.** Western blot analysis of TALE production in various *Xoo* strains. (A) Plasmids pHZWpthXo1, pHZWavrXa7 and pHZWavrXa27 were transferred into *Xoo* strains PH and PE by electroporation. Production of TALEs was analyzed by western blotting using an anti-FLAG primary antibody (see Methods). (B) Plasmid pHZWpthXo7 was transferred into *Xoo* strain GX4 by electroporation, and production of PthXo7 was detected using an anti-FLAG antibody. M, molecular weight marker (kDa).
