# Supplementary material for: Xanthomonas oryzae pv. oryzae TALE proteins recruit OsTFIIAγ1 to compensate for the absence of OsTFIIAγ5 in bacterial blight in rice
Source: Mol Plant Pathol. 2018 Aug 7;19(10):2248–62. doi: 10.1111/mpp.12696 (PMC6638009; doi:10.1111/mpp.12696)
Supplement: Supplementary file 4 — Fig. S4 Modification of OsTFIIAγ1 in IRBB5 rice by Clustered regularly interspaced short palin dromic repeats and CRISPR‐associated protein 9 editing. [file MPP-19-2248-s004.docx]

**
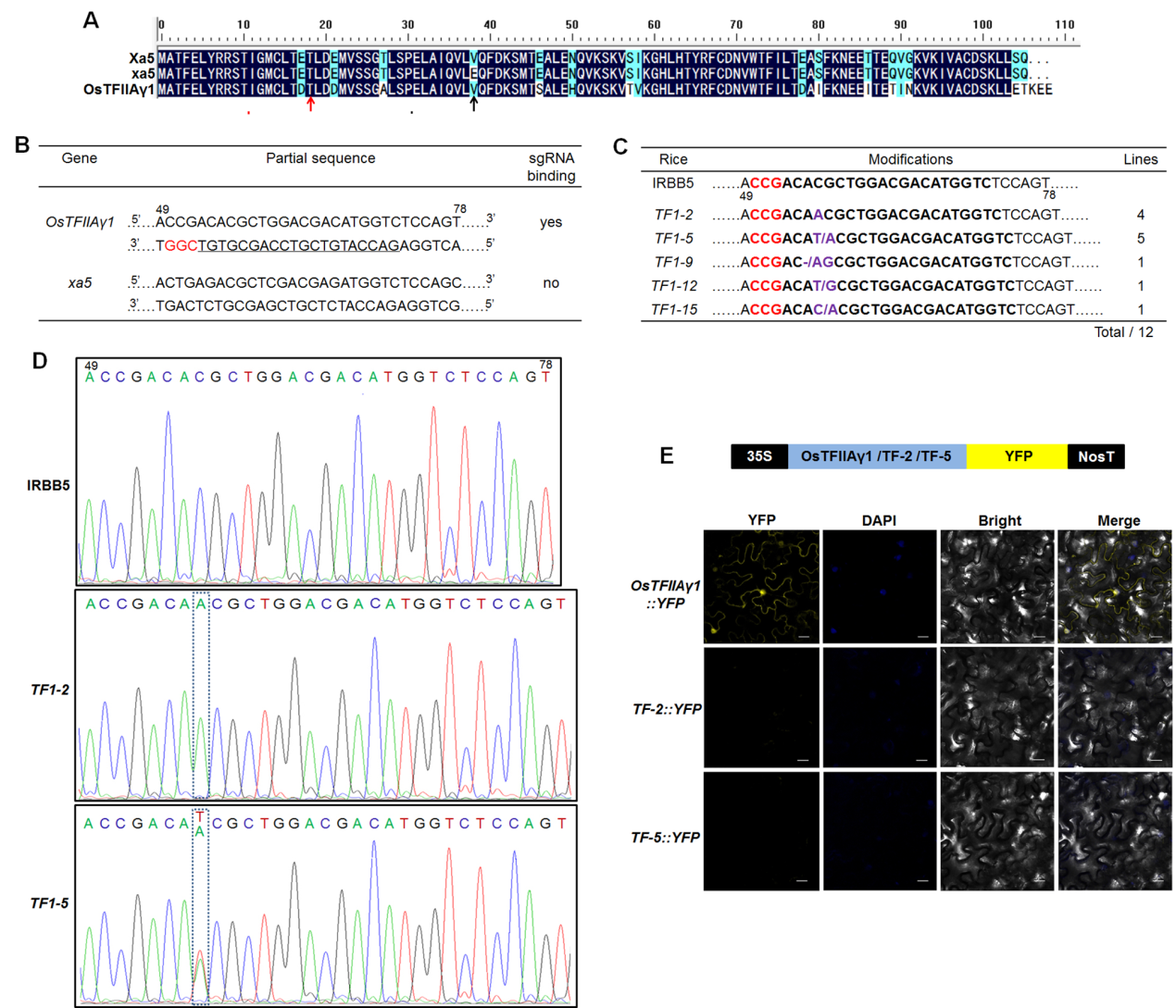
**

**Figure S4.** Modification of *OsTFIIAγ1* in the IRBB5 rice by CRISPR/Cas9 editing. (A) Alignment of Xa5, xa5 and OsTFIIAγ1 protein sequences. The red arrow indicates the amino acid residue that was mutated in *TF1-2* and *TF1-5* rice lines. The black arrow indicates the conserved valine (V) residue of in Xa5 and OsTFIIAγ1 and the mutated glutamate (E) in xa5. (B) Sequence of the 20-bp region targeted for Cas9/sgRNA editing in the first exon of *OsTFIIAγ1* (see underscored nucleotides). The protospacer adjacent motif (PAM) for cleavage is indicated in red font. The sequence of the corresponding region in *xa5* is shown below. (C) Genomic DNA sequence of the target region in IRBB5 and five edited rice lines (*TF1-2, TF1-5, TF1-9, TF1-12*, and *TF1-15*). Cas9/sgRNA-mediated DNA cleavage and subsequent error-prone repair in the five edited lines resulted in mutant forms of *OsTFIIAγ1* due to insertions (letters in purple font) or deletions (dashes in purple). Column on the right shows the number of rice lines with the described mutation. A total of 12 lines were generated, and lines *TF1*-2 and *TF1*-5 were chosen for further analysis. (D) Sequence of nucleotides 49-78 in *OsTFIIAγ1* amplicons generated from rice lines IRBB5, *TF1-2* and *TF1-5*. The rectangles (see dashed line) indicate the nucleotide insertions in *TF1*-2 and *TF1*-5. The two peaks at the insertion site in *TF1*-5 represent different insertions in two chromosomes. (E) Assay for *OsTFIIAγ1* functionality using fluorescence microscopy. The functional map of the constructs used in this experiment is shown. The CaMV 35S promoter was used to drive expression, and the three genes were fused in frame to YFP; the nopaline synthase termination signal is shown (NosT). OsTFIIAγ1::YFP, TF-2::YFP*,* and TF-5::YFP fusions were transiently expressed in tobacco using *Agrobacterium*-mediated transformation, and designated as OsTFIIAγ1::YFP, TF-2::YFP, and TF-5::YFP, respectively. Images of cells expressing fused YFP were taken at 48 hpi. DAPI was used as a nuclear stain.
