# Supplementary material for: Xanthomonas oryzae pv. oryzae TALE proteins recruit OsTFIIAγ1 to compensate for the absence of OsTFIIAγ5 in bacterial blight in rice
Source: Mol Plant Pathol. 2018 Aug 7;19(10):2248–62. doi: 10.1111/mpp.12696 (PMC6638009; doi:10.1111/mpp.12696)
Supplement: Supplementary file 5 — Fig. S5 The affinity of PthXo1, AvrXa7 and AvrXa27 for Xa5, xa5 and OsTFIIAγ using microscale thermophoresis (MST). [file MPP-19-2248-s005.docx]

**
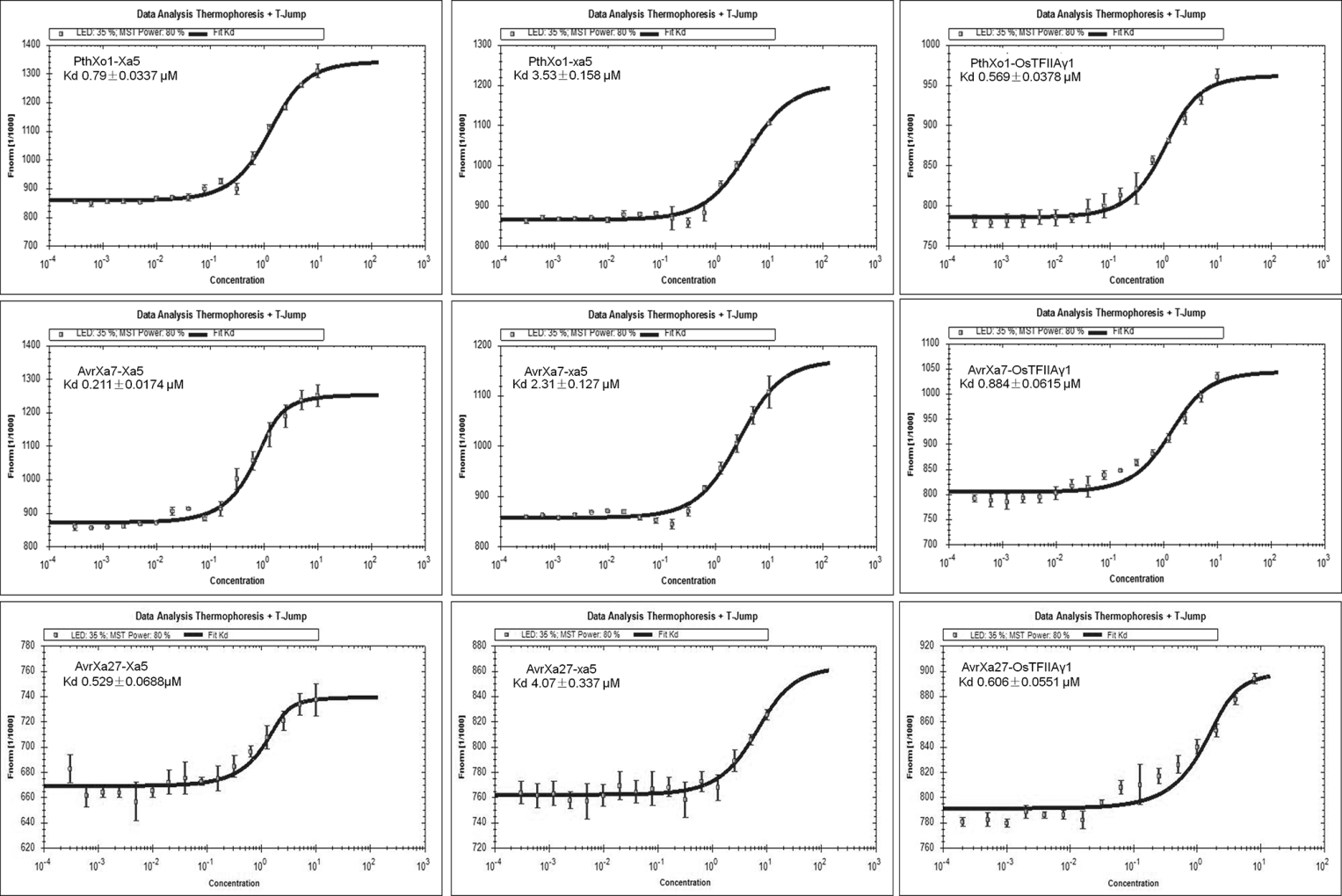
Figure S5.** The affinity of PthXo1, AvrXa7, and AvrXa27 for Xa5, xa5, and OsTFIIAγ using microscale thermophoresis (MST). The six genes were cloned in pET30a, overproduced as His-tagged proteins, and purified Xa5, xa5, and OsTFIIAγ were labeled with the amine-reactive, red fluorescent dye NT-647 as described in Methods. Labeled proteins (1 μM) were mixed with 16 different TALE concentrations (see Methods). Protein mixtures were incubated for 10 min at room temperature, and then loaded into silica capillaries. Measurements were performed at 25 °C using 35% LED power and 80% IR-laser power. MST was performed with a Monolith NT.115T (NanoTemper Technologies). NTAnalysis v. 1.5.41 was used to plot thermophoresis signals vs. ligand concentrations; dissociation constants (μM) were plotted for the nine interactions in Fig. 7B.
