# Supplementary material for: Xanthomonas oryzae pv. oryzae TALE proteins recruit OsTFIIAγ1 to compensate for the absence of OsTFIIAγ5 in bacterial blight in rice
Source: Mol Plant Pathol. 2018 Aug 7;19(10):2248–62. doi: 10.1111/mpp.12696 (PMC6638009; doi:10.1111/mpp.12696)
Supplement: Supplementary file 6 — Fig. S6 Xanthomonas oryzae pv. oryzae (Xoo) strain GX4 induces the expression of OsSWEET14, but not OsSWEET11, in IR24 rice. [file MPP-19-2248-s006.docx]

**
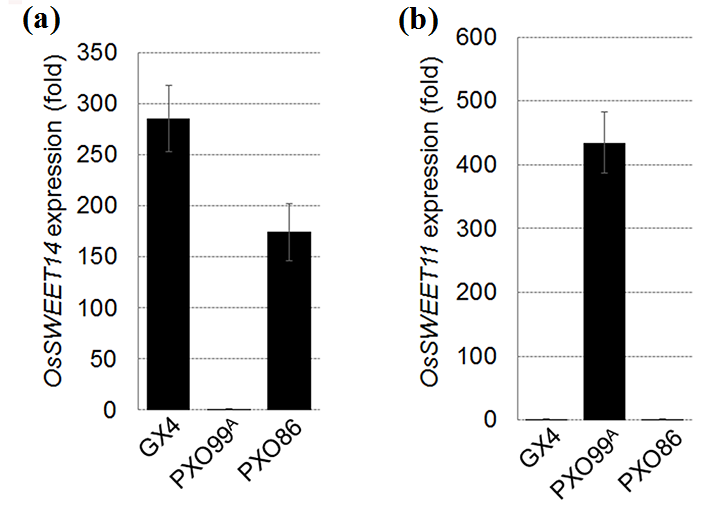
**

**Figure S6**. *Xoo* strain GX4 induces the expression of *OsSWEET14* (A) but not *OsSWEET11* (B) in IR24 rice. *Xoo* GX4, PX099^A^, and PXO86 were inoculated to IR24 rice and the expression of *OsSWEET14* and *OsSWEET11* was evaluated by qRT-PCR at 24 hpi.
