# Supplementary material for: Xanthomonas oryzae pv. oryzae TALE proteins recruit OsTFIIAγ1 to compensate for the absence of OsTFIIAγ5 in bacterial blight in rice
Source: Mol Plant Pathol. 2018 Aug 7;19(10):2248–62. doi: 10.1111/mpp.12696 (PMC6638009; doi:10.1111/mpp.12696)
Supplement: Supplementary file 7 — Methods S1 DNA manipulation and plasmid construction. [file MPP-19-2248-s007.docx]

**Methods S1. DNA manipulation and plasmid construction.**

Plasmid Miniprep and DNA Gel Extraction kits were purchased from Axygen (Beijing, China). Restriction endonucleases, DNA ligases, Ex Taq DNA polymerase, and DNA molecular weight markers were provided by TaKaRa (Dalian, China). Total RNA kits were supplied by TransGen Biotech (Beijing). Primers were synthesized by Generay (Shanghai).

Plasmid pZWavrXa7, which was kindly supplied by Dr. Bing Yang, was used to construct plasmids for expression of *tal* genes in *Xoo* strains. The construct pZWavrXa7 was digested with *Sph*I to release *avrXa7* and replaced with *avrXa27*, *pthXo1* and *pthXo7* to create pZWavrXa27, pZWpthXo1 and pZWpthXo7, respectively (Supplementary Information, Table S1). Plasmids pZWavrXa7, pZWavrXa27, pZWpthXo1 or pZWpthXo7 were then fused with the broad-host-range vector pHM1 at the *Hin*dIII site to generate pHZWavrXa7, pHZWavrXa27, pHZWpthXo1 and pHZWpthXo7, respectively. Constructs were introduced into *Xoo* strains by electroporation (2.5 kv, 4 ms).
