# Supplementary material for: Xanthomonas oryzae pv. oryzae TALE proteins recruit OsTFIIAγ1 to compensate for the absence of OsTFIIAγ5 in bacterial blight in rice
Source: Mol Plant Pathol. 2018 Aug 7;19(10):2248–62. doi: 10.1111/mpp.12696 (PMC6638009; doi:10.1111/mpp.12696)
Supplement: Supplementary file 8 — Methods S2 Immunoblotting assays. [file MPP-19-2248-s008.docx]

**Methods S2. Immunoblotting assays.**

Western blot analysis with FLAG-labeled antisera was used to detect the production of TALE proteins PthXo1, AvrXa7, AvrXa27 and PthXo7 in *Xoo* strains. Construct pZWavrXa7 contained *avrXa7* in-frame with C-terminal FLAG epitopes (Table S1). The *SphI* fragment of *avrXa7* was replaced with *avrXa27, pthXo1* and *pthXo7* to create pZWavrXa27, pZWpthXo1 and pZWpthXo7, which were then ligated into the broad-host range vector pHM1 at the *Hin*dIII site. The resulting constructs, pHZWavrXa27, pHZWpthXo1, pHZWpthXo7, and pHZWavrXa7 were then transferred into *Xoo* strains.

Western blot analysis was used to detect the production of TALEs in *Xoo* strains. Briefly, *Xoo* strains were cultured in NB to the logarithmic phase and harvested by centrifugation. Bacterial cells were washed twice, and adjusted to OD_600_ = 1.0 with sterile distilled water. SDS loading buffer (5X) was added to the bacterial suspensions and boiled in a water bath for 10 min. Proteins were separated on 8% SDS-PAGE gels and transferred to polyvinylidene difluoride membranes for immunoblotting using anti-FLAG (TransGen, Beijing, China) as the primary antibody. Primary antibodies were detected using goat anti-rabbit IgG (H + L) (TransGen) and visualized with the EasySee Western Kit (TransGen).
