# Supplementary material for: Xanthomonas oryzae pv. oryzae TALE proteins recruit OsTFIIAγ1 to compensate for the absence of OsTFIIAγ5 in bacterial blight in rice
Source: Mol Plant Pathol. 2018 Aug 7;19(10):2248–62. doi: 10.1111/mpp.12696 (PMC6638009; doi:10.1111/mpp.12696)
Supplement: Supplementary file 9 — Methods S3 Assembly of yellow fluorescent protein (YFP)‐tagged constructs. [file MPP-19-2248-s009.docx]

**Methods S3. Assembly of YFP-tagged constructs.**

Selected *tal* genes (*pthXo1*, *avrXa7*, and *avrXa27*) were introduced into the C-terminus of YFP (YC) using the restriction sites *Xba*I and *Sma*I. Difficulties were encountered in attempting to amplify full-length *tal* genes using PCR; therefore, we amplified the N- and C-terminus of each *tal* using primer pairs tal-F(*Xba*I)/tal-N-R(*Sph*I) and tal-C-F(*Sph*I)/tal-R(*Sma*I), respectively (Table S2). The products from PCR were combined by ligating the *Sph*I sites, and the N- and C-terminal portions of each *tal* were cloned into YC vector as *Xba*I/*Sma*I fragments. Finally, the conserved *Bam*HI fragment of each *tal* was inserted between the N and C-terminal regions of each gene, resulting in pthXo1::YC, avrXa7::YC, and avrXa27::YC. The YN and YC constructs were then transformed into *Agrobacterium* strain GV3101, and used for transient expression in *N. benthamiana* as described above.

To investigate whether the *TF-2* and *TF-5* genes were expressed as functional proteins, we cloned *OsTFIIAγ1, TF-2* and *TF-5* as N-terminal translational fusions to yellow fluorescent protein (YFP), resulting in OsTFIIAγ1::YFP, TF-2::YFP, and TF-5::YFP, respectively. The vector containing full-length YFP was constructed by fusing the N- and C-terminal portions of YFP using pSPYNE and pSPYCE (Table S1) ([Walter *et al.*, 2004](#_ENREF_3)). Briefly, the C-terminal end of the YFP coding sequence was amplified from pSPYCE with primer pairs YC-F/Nos-R (Table S2) and then ligated into pSPYNE using the One Step Cloning Kit (Vazyme Biotech, China). The resulting recombinant plasmid was designated as pYFP. *OsTFIIAγ1, TF-2* and *TF-5* were amplified using primer pairs TFIIAγ1-YN-F(*Xba*I)/TFIIAγ1-YN-R(*Sma*I) (Table S2) from rice lines IRBB5, *TF1*-2 and *TF1*-5, respectively, and then ligated into pYFP using the *Xba*I and *Sma*I. The recombinant plasmids were transferred into *Agrobacteria* GV3101.

For transient expression in tobacco, *Agrobacteria* GV3101 strains containing YFP constructs were cultured to OD_600_=1.5, harvested by centrifugation, and re-suspended in inducing buffer (10 mM MgCl_2_, 0.2 mM acetosyringone and 200 mM MES, pH 5.6) to OD_600_=1.0. Buffer-supplemented *Agrobacterium* strains containing the individual YFP constructs (OsTFIIAγ1::-YFP, TF-2::YFP, and TF-5::YFP, see Table S1) were mixed at a ratio of 1:1 and incubated at 25 °C for 1 h. The induced *Agrobacterium* mixtures were infiltrated into *Nicotiana benthamiana* (tobacco) leaves. After 48 hpi, fluorescence was imaged with a confocal laser fluorescence microscope, and 4′, 6-diamidino-2-phenylindole (DAPI, 100 μg/ml) was used for nuclear staining.

**Walter, M., Chaban, C., Schutze, K., Batistic, O., Weckermann, K., Nake, C.*, et al.* (2004) Visualization of protein interactions in living plant cells using bimolecular fluorescence complementation. *The Plant journal : for cell and molecular biology,* 40, 428-438.**
