# Supplementary material for: Xanthomonas oryzae pv. oryzae TALE proteins recruit OsTFIIAγ1 to compensate for the absence of OsTFIIAγ5 in bacterial blight in rice
Source: Mol Plant Pathol. 2018 Aug 7;19(10):2248–62. doi: 10.1111/mpp.12696 (PMC6638009; doi:10.1111/mpp.12696)
Supplement: Supplementary file 10 — Methods S4 Protein production and purification. [file MPP-19-2248-s010.docx]

**Methods S4. Protein production and purification.**

For overproduction of proteins in *E. coli*, genes were amplified and then cloned into pET30a. *OsTFIIAγ1* was amplified using primers TFIIAγ1-30a-F(*Nco*I) and TFIIAγ1-30a-R(*Xho*I) and ligated into *Nco*I/*Xho*I-digested pET30a. *Xa5* and *xa5* were amplified using primer sets Xa5-30a-F(*Bam*HI) and Xa5-30a-R(*Xho*I) and ligated into *Bam*HI/*Xho*I-digested pET30a. Due to their size, we used a multi-step process to clone *avrXa7*, *avrXa27*, and *pthXo1* in pET30a. The N- and C-terminal regions were amplified using primer pairs tal-F(*Eco*RI)/tal-N-R(*Sph*I) and tal-C-F(*Sph*I)/tal-R(*Xho*I), respectively. These PCR products were cloned in *Eco*RI/*Xho*I-digested pET30a. Finally, we excised the central portion of *avrXa7*, *avrXa27*, and *pthXo1* in constructs pZWavrXa7, pZWavrXa27, and pZWpthXo1, respectively. The central portion of each *tal* was then ligated these between the N- and C-terminal segments at conserved *Not*I and *Sal*I sites. This final cloning step reconstructed *avrXa7*, *avrXa27*, and *pthXo1* in pET30a, resulting in pET30a-avrXa7, pET30a-avrXa27, and pET30a-pthXo1, respectively. The constructs and primers used for cloning in pET30a are described in Tables S1 and S2, respectively.

Recombinant TALE and OsTFIIAγ in pET30a were overproduced in *E. coli* BL21(DE3) in LB broth as described previously with slight modifications ([Zou *et al.*, 2012](#_ENREF_5)). Recombinant proteins were induced for 16 h with 0.5 mM IPTG at 16^o^C. The induced bacterial cells (300 ml) were harvested, disrupted by sonication, and the supernatant was applied to Ni-NTA His-Bind resin as recommended by the manufacturer (Novagen). His_6_-fused proteins were concentrated using Centricon YM-30 columns (Millipore); the elute buffer used for elution was exchanged with storage buffer (50 mM Tris-HCl pH 8.0, 0.5 mM EDTA, 50 mM NaCl and 5% glycerol), and proteins were stored at -80 ℃ until needed.

**Zou, H. S., Song, X., Zou, L. F., Yuan, L., Li, Y. R., Guo, W.*, et al.* (2012) EcpA, an extracellular protease, is a specific virulence factor required by Xanthomonas oryzae pv. oryzicola but not by X. oryzae pv. oryzae in rice. *Microbiology+,* 158, 2372-2383.**
