# Supplementary material for: Xanthomonas oryzae pv. oryzae TALE proteins recruit OsTFIIAγ1 to compensate for the absence of OsTFIIAγ5 in bacterial blight in rice
Source: Mol Plant Pathol. 2018 Aug 7;19(10):2248–62. doi: 10.1111/mpp.12696 (PMC6638009; doi:10.1111/mpp.12696)
Supplement: Supplementary file 11 — Table S1 Strains and plasmids used in this study. [file MPP-19-2248-s011.docx]

**Table S1.** Strains and plasmids used in this study.

| Strain or Plasmid | Description | Reference or Source | |
| --- | --- | --- | --- |
| Strains |  |  | |
| *Escherichia coli* | | | |
| DH5α | F^-^ *endA1*, *thi-1*, *recA1*, Φ80*lacZ*, ΔM15 | This lab | |
| BL21(DE3) | F*^-^ ompT, hsdS20, gal* | This lab | |
| *Agrobacterium tumefaciens* | | | |
| *GV3101* | C58, Ti pMP90(pTiC58ΔT-DNA), Rif^r^ | This lab | |
| *X. oryzae pv. oryzae* | | | |
| PXO99^A^ | Wild-type, Philippine race 6; Rif^r^ | This lab | |
| PXO86 | Wild-type, Philippine race 2; Rif^r^ | This lab | |
| PH | Derived from PXO99^A^, *tal*-free strain | ([Ji *et al.*, 2016](#_ENREF_2)) | |
| PE | Derived from PXO99^A^, *tal* deletion mutant that lacks *tal2*, *tal3*, *tal5*, *tal7*, *tal8* and *tal9* clusters | ([Ji et al., 2016](#_ENREF_2)) | |
| PH(*pthXo1*) | PH with pHZWpthXo1 | This study | |
| PE(*pthXo1*) | PE with pHZW pthXo1 | This study | |
| PH(*avrXa7*) | PH with pHZWavrXa7 | This study | |
| PE(*avrXa7*) | PE with pHZWavrXa7 | This study | |
| PH(*avrXa27*) | PH with pHZWavrXa27 | This study | |
| PE(*avrXa27*) | PE with pHZWavrXa27 | This study | |
| GX4 | Wild-type, China isolate | This lab | |
| GX4(*pthXo7*) | GX4 with pHZWpthXo7 | This study | |
| XZ44 | Wild-type, China isolate | This lab | |
| JS-97-2 | Wild-type, China isolate | This lab | |
| YC5 | Wild-type, China isolate | This lab | |
| AH28 | Wild-type, China isolate | This lab | |
| KS-153-1 | Wild-type, China isolate | This lab | |
| KS-3-8 | Wild-type, China isolate | This lab | |
| KS-39-4 | Wild-type, China isolate | This lab | |
| Plasmids |  |  | |
|  |  |  | |
| pHM1 | Broad-spectrum cosmid vector, Sp^r^ | ([Hopkins *et al.*, 1992](#_ENREF_1)) | |
| pET30a | pBR322 origin, F1 origin, *lacI*, His-Tag, S-Tag, Km^r^ | Novagen | |
| pZWavrXa7 | *avrXa7* in pBluescript II KS+, contains FLAG epitope immediately downstream of the second *Sph*I site in the C-terminus of AvrXa7, Ap^r^ | ([Yang *et al.*, 2000](#_ENREF_4)) | |
| pHZWavrXa7 | pHM1 fused with pZWavrXa7 at *Hin*dIII site; *lacZ* promoter upstream of *avrXa7*, Ap^r^, Sp^r^ | ([Yang et al., 2000](#_ENREF_4)) | |
| pZWavrXa27 | *Sph*I fragment of *avrXa27* in pZWavrXa7; Ap^r^ | This study | |
| pHZWavrXa27 | pHM1 fused with pZWavrXa27 at *Hin*dIII, *lacZ* promoter upstream of *avrXa27*, Ap^r^, Sp^r^ | This study | |
| pZWpthXo1 | *Sph*I fragment of *pthXo1* in pZWavrXa7, Ap^r^ | This study | |
| pHZWpthXo1 | pHM1 fused to pZWpthXo1 at *Hin*dIII site, *pthXo1* fused with *lacZ* promoter, Ap^r^, Sp^r^ | This study | |
| pZWpthXo7 | *Sph*I fragment of *pthXo7* in pZWavrXa7 | This study | |
| pHZWpthxo7 | pHM1 fused with pZWpthXo7 using *Hind*III, *pthXo7* fused with *lacZ* promoter, Ap^r^, Sp^r^ | This study | |
| pET30a-avrXa7 | *avrXa7* in pET30a, Km^r^ | This study | |
| pET30a-avrXa27 | *avrXa27* in pET30a, Km^r^ | This study | |
| pET30a-pthXo1 | *pthXo1* in pET30a, Km^r^ | This study | |
| pET30a-XA5 | *Xa5* in pET30a, Km^r^ | This study | |
| pET30a-xa5 | *xa5* in pET30a, Km^r^ | This study | |
| pET30a-OsTFIIAγ1 | *OsTFIIAγ1* in pET30a, Km^r^ | This study | |
| pYFP | Binary vector with full-length YFP coding gene, Km^r^ | This study | |
| OsTFIIAγ1-YFP | Translational fusion, OsTFIIAγ1::YFP, Km^r^ | This study | |
| TF1-2-YFP | Translational fusion, TF1-2::YFP with YFP, Km^r^ | This study | |
| TF1-5-YFP | Translational fusion, TF1-5::YFP, Km^r^ | This study | |
| pSPYNE | Binary vector with N terminal of YFP, Km^r^ | ([Walter et al., 2004](#_ENREF_3)) | |
| pSPYCE | Binary vector with C terminal of YFP, Km^r^ | ([Walter et al., 2004](#_ENREF_3)) | |
| Xa5::YN | *Xa5* in pSPYNE , Km^r^ | This study | |
| xa5::YN | *xa5* in pSPYNE, Km^r^ | This study | |
| OsTFIIAγ1::YN | *OsTFIIAγ1* in pSPYNE, Km^r^ | This study | |
| PthXo1::YC | *pthXo1* in pSPYCE, Km^r^ | This study | |
| AvrXa7::YC | *avrXa7* in pSPYCE, Km^r^ | This study |  |
| AvrXa27::YC | *avrXa27* in pSPYCE, Km^r^ | This study |  |

Ap^r^, Sp^r^, Km^r^, and Rif^r^ indicate resistance to spectinomycin, ampicillin, kanamycin and rifampicin, respectively.

**Hopkins, C. M., White, F. F., Choi, S. H., Guo, A. and Leach, J. E. (1992) Identification of a family of avirulence genes from Xanthomonas oryzae pv. oryzae. *Molecular plant-microbe interactions : MPMI,* 5, 451-459.**

**Ji, Z., Ji, C., Liu, B., Zou, L., Chen, G. and Yang, B. (2016) Interfering TAL effectors of Xanthomonas oryzae neutralize R-gene-mediated plant disease resistance. *Nature communications,* 7, 13435.**

**Walter, M., Chaban, C., Schutze, K., Batistic, O., Weckermann, K., Nake, C.*, et al.* (2004) Visualization of protein interactions in living plant cells using bimolecular fluorescence complementation. *The Plant journal : for cell and molecular biology,* 40, 428-438.**

**Yang, B., Zhu, W., Johnson, L. B. and White, F. F. (2000) The virulence factor AvrXa7 of Xanthomonas oryzae pv. oryzae is a type III secretion pathway-dependent nuclear-localized double-stranded DNA-binding protein. *Proceedings of the National Academy of Sciences of the United States of America,* 97, 9807-9812.**
