# Supplementary material for: Xanthomonas oryzae pv. oryzae TALE proteins recruit OsTFIIAγ1 to compensate for the absence of OsTFIIAγ5 in bacterial blight in rice
Source: Mol Plant Pathol. 2018 Aug 7;19(10):2248–62. doi: 10.1111/mpp.12696 (PMC6638009; doi:10.1111/mpp.12696)
Supplement: Supplementary file 12 — Table S2 Primers used in this study. [file MPP-19-2248-s012.docx]

**Table S2.** Primers used in this study.

| Primer name | Sequence |
| --- | --- |
| OsActin qRT-F | 5′-GTTCCTGCTGTTTGTTCTGTTG-3′ |
| OsActin qRT-R | 5′-ATCTCACGCATTACCCTACCTT-3′ |
| OsSWEET11-qRT-F | 5′-AGTCGACGGGAGGGTACAG-3′ |
| OsSWEET11-qRT-R | 5′-TGATGGTCAGCAGCGGC-3′ |
| OsSWEET14-qRT-F | 5′-TCTACGCCCCCAAGAAGGCCA-3′ |
| OsSWEET14-qRT-R | 5′-ACCCAACCAAGAACCACGATGC-3′ |
| Xa27-qRT-F | 5′-CTCGCCATGCTGTCGCTCGT-3′ |
| Xa27-qRT-R | 5′-TAGAGAGACCAGAGACCACCAAGCA-3′ |
| TFIIAγ1-qRT-F | 5′-TGACAAGTCCATGACTAGC-3′ |
| TFIIAγ1-qRT-R | 5′-ATCTCTTCGTTCTTGAAAAT-3′ |
| Xa5-qRT-F | 5′-TGATAAGTCTATGACGGAA-3′ |
| Xa5-qRT-R | 5′-CTGTAGTCTCCTCGTTCTTGAATGA-3′ |
| TFIIAγ1-YN-F (*Xba*I) | 5′-TCTAGA ATGGCCACCTTCGAGCTG -3′ |
| TFIIAγ1-YN-R (*Sma*I) | 5′-CCCGGG CTCTTCTTTAGTCTCCAGCAA-3′ |
| Xa5-YN-F (*Xba*I) | 5′-TCTAGA ATGGCCACCTTCGAGCTCTACCGGA-3′ |
| Xa5-YN-R (*Sma*I) | 5′-CCCGGG TTGGCTGAGTAGTTTGGAATC-3′ |
| tal-F (*Xba*I) | 5′-TCTAGA ATGGATCCCATTCGTTCGC-3′ |
| tal-R (*Sma*I) | 5′-TCCCCCGGG GATCGTCCCTCCGACTGAGCC-3 |
| tal-N-R (*Sph*I) | 5′-TGCATTGCGCCAT GCATGC ACTGCCTC-3′ |
| tal-C-F(*Sph*I) | 5′-ATCAGGCGTCTTT GCATGC ATTCGCCGA-3′ |
| TFIIAγ1-30a-F (*Nco*I) | 5′-CCATGG CCACCTTCGAGCTG-3′ |
| TFIIAγ1-30a-R(*Xho*I) | 5′-CTCGAG CTCTTCTTTAGTCTCCAGCAA-3′ |
| Xa5-30a-F (*Bam*HI) | 5′-GGATCC ATGGCCACCTTCGAGCTCTAC-3′ |
| Xa5-30a-R (*Xho*I) | 5′-CTCGAG TTGGCTGAGTAGTTTGGAAT-3′ |
| tal-F (*EcoR*I) | 5′-ACA GAATTC ATGGATCCCATTCGTTCGCG-3′ |
| tal-R (*Xho*I) | 5′-AAA CTCGAG TCAGATCGTCCCTCCGACTGA-3′ |
| TFIIAγ1-test-R | 5′-GTGAGTAGCCTATGAAGCAAGCAG-3′ |
| YC-F | 5′-GTCTATATCATGGCCGACAAGCAGAAGAACGGCATC-3′ |
| Nos-R | 5′-GTTTGAACGATCGGGGAAATTC-3′ |

The endonuclease recognition sites are underscored.
